# Synthesis and Improved Cross-Linking Properties of C5-Modified Furan Bearing PNAs

**DOI:** 10.3390/molecules22112010

**Published:** 2017-11-20

**Authors:** Joke Elskens, Alex Manicardi, Valentina Costi, Annemieke Madder, Roberto Corradini

**Affiliations:** 1 Organic and Biomimetic Chemistry Research Group, Department of Organic and Macromolecular Chemistry, Ghent University, Krijgslaan 281-S4, 9000 Gent, Belgium; Joke.Elskens@Ugent.be (J.E.); Alex.Manicardi@Ugent.be (A.M.); 2 Department of Chemistry, Life Sciences and Environmental Sustainability, University of Parma, Parco Area delle Scienze 17/A, 43124 Parma, Italy; valentina.costi@studenti.unipr.it

**Keywords:** PNA, backbone modification, furan, crosslinking, chiral monomers

## Abstract

Over the past decades, peptide nucleic acid/DNA (PNA:DNA) duplex stability has been improved via backbone modification, often achieved via introducing an amino acid side chain at the α- or γ-position in the PNA sequence. It was previously shown that interstrand cross-linking can further enhance the binding event. In this work, we combined both strategies to fine-tune PNA crosslinking towards single stranded DNA sequences using a furan oxidation-based crosslinking method; for this purpose, γ-l-lysine and γ-l-arginine furan-PNA monomers were synthesized and incorporated in PNA sequences via solid phase synthesis. It was shown that the l-lysine γ-modification had a beneficial effect on crosslink efficiency due to pre-organization of the PNA helix and a favorable electrostatic interaction between the positively-charged lysine and the negatively-charged DNA backbone. Moreover, the crosslink yield could be optimized by carefully choosing the type of furan PNA monomer. This work is the first to describe a selective and biocompatible furan crosslinking strategy for crosslinking of γ-modified PNA sequences towards single-stranded DNA.

## 1. Introduction

Peptide nucleic acids (PNAs), first described by Nielsen et al. [1], are a class of nuclease-resistant synthetic oligonucleotide mimics based on repeating *N*-(2-aminoethyl)-glycine units. The uncharged backbone of the PNA reduces electrostatic repulsion upon hybridization, resulting in very strong binding affinity towards complementary oligonucleotides (ONs) [2]. PNAs have various applications in antisense [3,4,5] and antigene strategies [6,7]. Furthermore, PNAs have been successfully used as artificial restriction DNA cutters [8,9] or artificial RNA restriction enzymes [10,11], as probes for mRNA detection [12,13], as anti-microRNA agents [14,15,16,17], and in microarray applications [18,19,20,21].

Further enhancement of oligonucleotide binding can be achieved by introducing modifications in the backbone structure of the PNA strand [22]. Backbone modification with amino acid side chains is one of the most reported and studied modifications of the original structure of the PNA monomer [23,24,25,26,27,28,29], of which γ-substitution is the most popular. Ly et al. reported on l-serine- and l-alanine-modified γ-PNA that show increased binding affinity towards DNA [30]; this was shown to be the result of a constrained conformation of the single strand, as recently demonstrated using molecular dynamics simulations [31], which leads to a lesser entropy loss in the formation of the PNA:DNA duplex. The use of γ-l-Lys-PNA building blocks has been reported as a synthetic handle for the attachment of various functional groups [32] including fluorophores and peptides. Additionally, guanidine based α- and γ-PNAs have been developed to improve cellular uptake [33,34,35]. Glazer et al. developed tail-clamp PNAs for gene editing purposes, featuring a polyethylene glycol γ-modification to improve water solubility without changing electrostatic interactions. These PNAs could be delivered in vivo via polymer nanoparticle formulation [36].

The high stability in the formation of PNA:ON duplexes might be advantageously combined with the use of interstrand crosslinking for therapeutic purposes. In 2006, Zhilina et al. reported on the development of PNA–nitrogen mustard conjugates, used for DNA alkylation and interstrand crosslinking towards the human epidermal growth factor receptor 2 (HER2/neu) promotor [37]. The use of 2-amino-6-vinylpurine as a crosslink moiety allowed selective crosslinking towards single-stranded DNA and RNA [38]. Alternatively, ‘switch-on’ crosslinking methods have been developed, where the pro-reactive moiety is activated upon an external trigger [39,40,41]. Also, crosslinking of PNA:DNA duplexes induced by γ-irradiation has been reported [42]. Unfortunately, these methodologies often require harsh reaction conditions [40,42], long reaction times [38], or impart problems like unspecific [37,42] and reversible [41] crosslinking, toxicity [39], and cellular damage [37,40,42]. In our research group, a furan oxidation-based crosslinking strategy was developed and successfully implemented for interstrand crosslinking of nucleic acids [43,44], crosslinking of DNA and RNA with proteins [45], and crosslinking of peptides with proteins [46] while ensuring a high selectivity and crosslinking yield. Furan oxidation can either be achieved via *N*-bromosuccinimide or via a biocompatible method using singlet oxygen. Recently, we also reported on the successful crosslinking of furan-modified peptide nucleic acids towards single- and double-stranded DNA [47]. Here, we became interested in further increasing the efficiency of furan–PNA crosslinking towards single-stranded DNA sequences, by exploiting the use of modified backbones.

## 2. Results and Discussion

### 2.1. Design and Synthesis of the Backbone-Modified *PNA* Monomers

In previous work we focused on the introduction of a furan moiety in the middle of a PNA strand to form an interstrand crosslink (ICL) with target ssDNA and dsDNA by means of the use of three different building blocks, where the furan unit was connected to the normal PNA backbone through three different geometries [47]. In the current study, we explore the possibility of introducing the furan modification in backbone-modified PNAs, in order to confer additional properties to the PNA strand, such as improved solubility, increased binding stability, and increased target selectivity by means of the introduction of positively-charged side chains. For this purpose, arginine and lysine side chains were selected as candidates for backbone modification, especially to try and compensate for the stability loss in the cases where the furan moiety was linked to the backbone and replaced the nucleobase unit, thus leading to a loss of the Watson–Crick base pairing ability.

C5(γ)-modified-PNA backbones derived from l-amino acids were selected over the C2(α)-modified ones (from d-amino acids) in view of the higher stability of the resulting duplexes with target DNA and RNA [21,35]. Moreover, unlike in the case of C2 modification, C5-modified PNA derivatives can be coupled to the PNA chain without extensive risk of epimerization, thus avoiding the need to apply submonomer-based strategies, earlier developed for C2 derivatives [48].

**Scheme 1:**
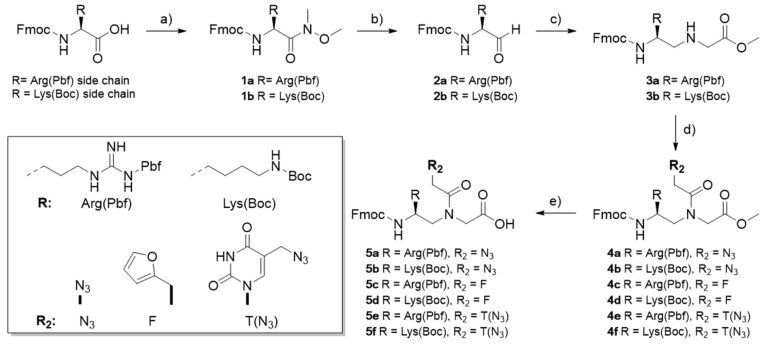
Synthesis of the 5L-modified monomers. (a) HBTU, DIPEA, *N*,*O*-dimethylhydroxylamine, DMF; (b) LiAlH_4_, THF; (c) Glycine methylester hydrochloride, NaBH_3_CN, AcOH, DIPEA, MeOH; (d) R_2_CH_2_COOH, EDC·HCl, DhBtOH, DIPEA, DMF; (e) Ba(OH)_2_, H_2_O:THF 1:1.

The synthesis of the required monomers for PNA synthesis, depicted in Scheme 1, starts from the commercially available amino acids Fmoc-Arg(Pbf) and Fmoc-Lys(Boc) which are converted to the corresponding Fmoc-protected aminoaldehydes **2a** and **2b** via reduction of the corresponding Weinreb amide (**1a** and **1b**, respectively). These aldehydes were then submitted to reductive amination in order to obtain the corresponding protected modified backbone building blocks **3a** and **3b**. EDC/DhBtOH coupling of the desired carboxylic acids followed by selective methyl ester deprotection allowed the synthesis of the desired monomers **5a**–**f**.

### 2.2. *PNA* Synthesis

**Figure 1:**
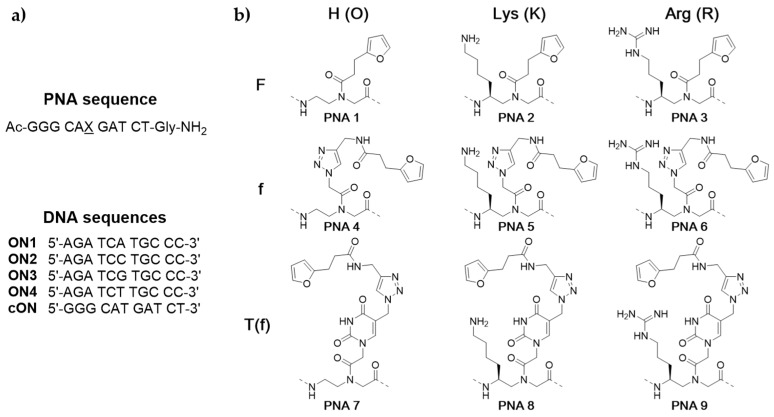
(**a**) Peptide nucleic acid (PNA) and DNA sequences used in this study; (**b**) Structure of the X monomers employed in this study with varying furan side- chain structure (**F**, **f**, **T**(**f**)) and varying backbone modification none (**O**), Lys side chain (**K**) or Arg side chain (**R**).

A series of six new PNAs were synthesized (see Figure 1, **PNA 2**–**3**, **PNA 5**–**6**, **PNA 8**–**9**); the sequence chosen for the study is identical to the one used in earlier work [47] and was selected for its complementarity to a specific region of the *MYCN* gene that was reported to be efficiently targeted for blocking gene expression [37]. The synthesis of the different PNA sequences was performed using a standard Fmoc/Bhoc solid phase synthesis protocol on Rink Amide ChemMatrix resin.

**Figure 2:**
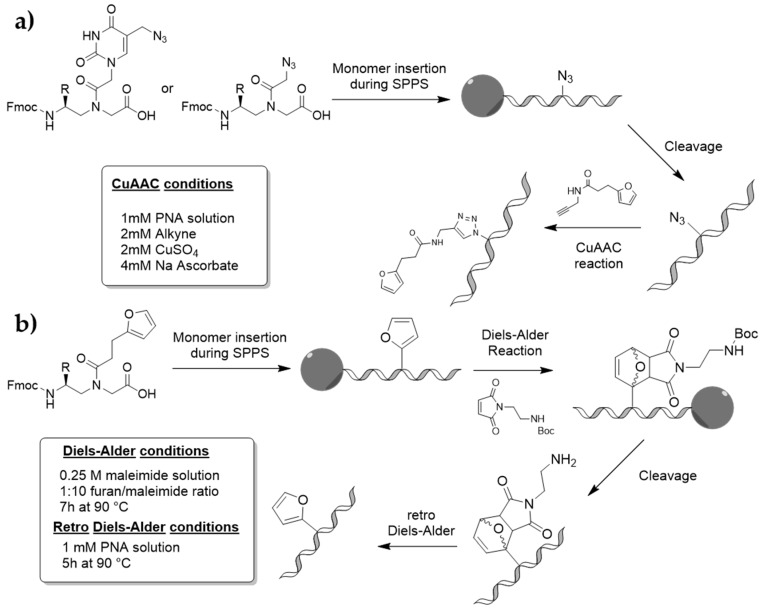
(**a**) General protocol for the synthesis of **PNA 4**–**9**; (**b**) general protocol for the synthesis of **PNA 1**–**3**. SPPS: solid phase peptide synthesis, CuAAC: Cu-catalyzed azide alkyn cyclodaddition.

In analogy with previous work, the synthesis of the **f** and **T**(**f**) series (see Figure 1, **PNA 5**–**6** and **PNA 8**–**9**) proceeded through incorporation of the different monomers bearing the azide function (designed starting from 2-azidoacetic acid for **PNA 5**–**6** or from 2-(5-azidomethyluracil-1-yl)acetic acid for **PNA 8**–**9**) in the growing chain. The full length PNAs were then cleaved from the resin and submitted to post-synthesis copper-catalyzed azide-alkyne cycloaddition reaction (CuAAC) in the presence of 3-(furan-2-yl)-*N*-(prop-2-yn-1-yl)propanamide (Figure 2a). For comparison the sequences of **PNA 1**, **4** and **7**, synthesized in a previous work [47], were used.

For the synthesis of the **F** series (**PNA 2**–**3**) we previously described that the furan unit must be protected from C2-alkylation by the benzhydrylic carbocation generated during the cleavage of the product from the resin [47]. A strategy exploiting a different cleavage cocktail mixture has already been proposed. However, this approach only allowed for mitigation of the formation of the undesired alkylated product but did not fully prevent it. We further found this procedure to be very sensitive to cleavage solution storage conditions (data not shown). For these reasons we decided to develop a protection protocol that had already been shown to allow efficient protection of maleimide residues during incorporation into oligonucleotides [49]. We here reasoned that a similar protection protocol might be applicable for the protection of the furan moiety during PNA synthesis. This approach is based on the temporary protection of the furan ring by on-resin formation of a [4 + 2] cycloadduct with maleimide, under thermal Diels–Alder (DA) conditions, and the subsequent retro-DA reaction in solution, after cleavage (Figure 2b). The chosen strategy relies on the actual possibility of tuning the DA/retro-DA conditions, by carefully choosing the maleimide *N*-substituents [50].

**Figure 3:**
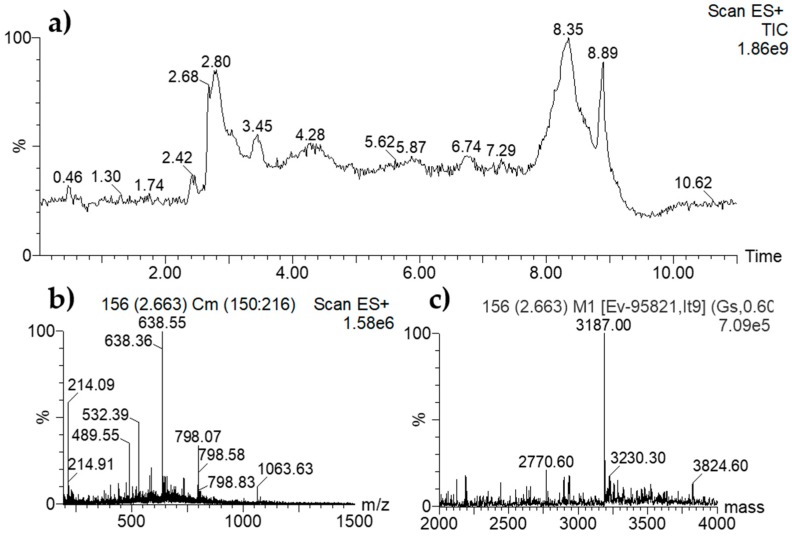
(**a**) UPLC-ESI-MS of crude **PNA 3** after the Diels–Alder (DA)/retro-DA; (**b**) MS trace of the 2.40–5.00 min region; and (**c**) de-convoluted spectrum from the MS trace (expected MW: 3187.8). The peak present in the 8.00–9.00 min region of the UPLC-MS corresponds to a column wash step.

The formation of the DA adduct was first optimized on a short PNA sequence, where it could be observed that the relative amount of maleimide, its concentration, and the time of reaction are crucial factors in achieving protection of the furan unit while avoiding the formation of unidentified by-products of reaction with the maleimide (see Supplementary Materials Figures S1–S3). Optimized conditions for the protection of the aromatic system from electrophilic substitution were obtained by prolongation of the DA reaction time (from 5 to 7 h) to ensure good DA-adduct formation with reduced by-product formation, and the use of a modified cleavage cocktail (10% m-cresol, 10% thioanisole in TFA) to avoid damage to any remaining unreacted furan. An example of the analysis of a time course experiment of the retro-DA reaction is shown in Figure S4. The optimized conditions were subsequently used for the more precious **PNA 1**–**3**, without isolating the intermediates. In Figure 3, a typical result obtained using this strategy is shown, where the 5 μmol scale resin bearing **PNA 3** was heated at 90 °C in the presence of a 0.25 M solution of *N*-(*N*-Boc-2-aminoethyl)maleimide in DMF (1:10 furan:maleimide ratio). The resulting resin was then cleaved and the crude was dissolved in 5 mL milliQ and heated for 5 h at 90 °C in order to induce the retro DA. As can be seen from the MS trace (Figure 3b) and from the de-convoluted spectrum (Figure 3c), only the signals relative to the desired product are present, and no significant formation of undesired products of alkylation (ΔMW: 166.2 Da) or overreaction with maleimide (ΔMW: 140.1 Da) were present.

### 2.3. Evaluation of *PNA:DNA* Duplex Stabilities

The effect of introduction of the backbone modification was studied through evaluation of the melting temperature of the complex formed between the synthesized PNA strands and the target DNA. All four base permutations facing the modification were studied (detailed sequence info for DNA strands used for the melting temperature (T_m_) evaluation are reported in Figure 1a). The results of these experiments are summarized in Table 1.

Analysis of the T_m_ data shows that in the **T**(**f**) series (**PNA 7**–**9**), where the base discrimination ability of the PNA is maintained, the introduction of the backbone modification (with the concurrent positive charge) is beneficial for the complex stability (cf. T_m_ for **PNA 8**–**9**/**ON 1**–**4** versus **PNA 7**/**ON 1**–**4**). There is no significant difference between Lys (**PNA 8**) versus Arg (**PNA 9**) side chains, suggesting that the different steric requirements of these two side chains do not play a crucial role in the complex formation. The stabilizing effect appears higher in the T:C (with **ON 2**) and T:G (with **ON 3**) mismatched sequences, where partial recognition of the nucleobases is still possible.

A similar stabilizing effect was also observed for the **f** series (cf. T_m_ for **PNA 5**–**6**/**ON 1**–**4** versus **PNA 4**/**ON 1**–**4**), where significant differences in stability are found between the different nucleobases, which are however similar for PNA with either the Lys or the Arg side chain. In previous studies, it was already hypothesized that the longer linker present in the **f** building block allowed the aromatic ring to interact with the nucleobases through π-π interaction. This interaction can force the furan ring to remain confined inside the helical duplex, while pointing the side chains toward the solvent.

Surprisingly, in the **F** series (cf. T_m_ for **PNA 2**–**3**/**ON 1**–**4** versus **PNA 1**/**ON 1**–**4**) the introduction of the positively charged side chain is not always beneficial for the complex stability. In this context the insertion of the bulkier and less flexible arginine side chain modification leads to a destabilization of the duplex, especially when a hindering purine nucleobase is placed in the complementary strand. In contrast, when a pyrimidine nucleobase is present, a small stabilization is observed. This behavior is not present when the lysine side chain is introduced. Taken together, these results suggest that the furan unit in the **F** building blocks adopts a conformation that can be perturbed by the introduction of the bulkier arginine side chain.

### 2.4. Evaluation of the Crosslink Reactivity of Furan–*PNAs* towards Single Stranded *DNA* Sequences

Next, the crosslink properties of the synthesized PNA sequences towards complementary single stranded DNAs **ON 1**–**4** were evaluated at a 10 μM strand concentration, using *N*-bromosuccinimide (1 equivalent every 15 min, 4 equivalents in total) to trigger the oxidation of the furan ring, and the results were analyzed through denaturing polyacrylamide gel experiments (PAGE).

**Figure 4:**
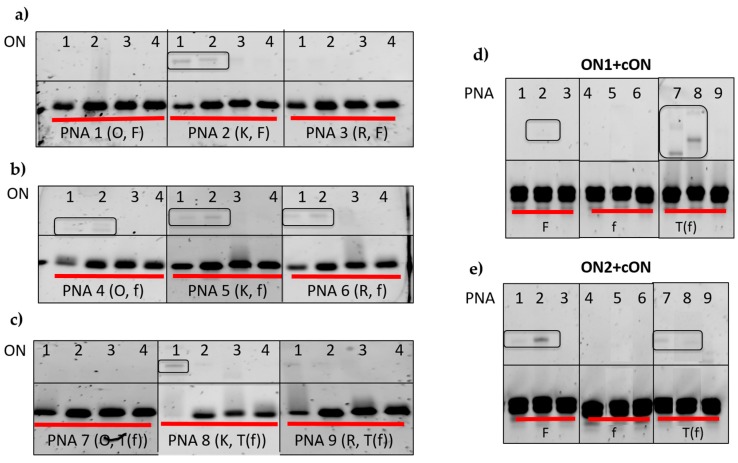
Denaturing polyacrylamide gel electrophoresis analysis of the crosslink experiments. (**a**) Crosslink experiment between **ON 1**–**4** and **PNA 1**–**3** (building block **F**); (**b**) Crosslink experiment between **ON 1**–**4** and **PNA 4**–**6** (building block **f**); (**c**) Crosslink experiment between **ON 1**–**4** and **PNA 7**–**9** (building block **T**(**f**)); (**d**) Strand displacement experiment between **ON 1**:**cON duplex** and **PNA 1**–**9**; (**e**) Strand displacement experiment between **ON 2**:**cON** duplex and **PNA 1**–**9**.

It can be seen in Figure 4 that when furan building block **F** (**PNA 1**–**3**) was used, crosslinking was most efficient when lysine-modified **PNA 2** was hybridized to **ON 1** (adenine) and **ON 2** (cytosine), resulting in the appearance of a slow mobility band on the gel (Figure 4a), with a light preference for the formation of the crosslink in presence of **ON 1**. In addition, with furan building block **f** (**PNA 4**–**6**), crosslinking seemed to be more efficient with the PNA strands containing the building block with the lysine side chain (**PNA 5**) and arginine modification (**PNA 6**), again with the selective formation of a slow migrating band only with **ON 1** and **ON 2**, but in this case with a small preference toward the cytosine counterpart (Figure 4b). Finally, in the case of furan building block **T (f)** (**PNA 7**–**9**), no crosslink is formed and the smearing of the bands which results in an apparent slower migration band (**PNA 8**:**ON 1** and **PNA 9**:**ON 1**) has to be attributed to the remaining PNA:DNA duplex which is not denatured under the analysis conditions. (Figure 4c). For experiments using target **ON 3**, bearing guanine opposite to the furan unit, only very faint bands (with **PNA 2** and **PNA 6**) and smears were observed; in none of the crosslink experiments with **ON 4** (bearing a thymine base) clearly visible slow mobility bands related to cross-linked species could be observed.

The results obtained in the **f** series (**PNA 4**–**6**) suggest that the introduction of a positive charge (**PNA 5**–**6**) in the PNA backbone is beneficial for the formation of the crosslink as the slow migration bands appears more intense compared to those obtained with **PNA 4**. Moreover, the migration difference observed in the different lanes is in line with the insertion of a positive charge in the ICL product. The identity of the ICL product was further confirmed by HPLC purification and MALDI analysis (see supplementary materials, Figure S16).

In contrast, in the **F** series (**PNA 1**–**3**) no ICL formation is observed when the arginine modification is introduced (**PNA 3**), possibly due to the fact that the bulkier modification is perturbing the orientation which the **F** monomer needs to adopt in order to react with the nucleophile placed on the opposed strand. This is also in line with the results obtained in the T_m_ evaluation, where the arginine side chain showed a destabilization effect on the duplex. The identity of the formed ICL product in the **PNA 2**–**ON 1** experiment was also confirmed by MALDI analysis (see Supplementary Materials, Figure S17). A schematic representation of the putative mechanism of ICL formation [51] for this series of PNA is depicted in Figure S18 (supplementary material).

For the **T**(**f**) series (**PNA 7**–**9**) no ICL was expected as the furan moiety is pointing in the major groove, and the introduction of the modification is only reflected in the degree of smearing of the band and appearance of a new band due to the PNA:DNA complexes of increased stability. Under these conditions, the most stable duplex appears to be that formed by the **PNA 8**, with the l-Lys side chain (Figure 4c).

Next, strand displacement experiments were performed. Since crosslinking only occurred with adenine or cytosine as complementary base (which is in line with previous results in dsDNA crosslinking), it was chosen to only perform these experiments with **ON 1** and **ON 2**. The DNA duplex was first allowed to form by annealing the two complementary strands, before the addition of the PNA strand. The solution was then left to equilibrate for 14 h at 37 °C before activation of the furan.

Results are shown in Figure 4d–e and confirm the results obtained in the initial screening experiments to determine the ability of the different PNA strands for ICL formation toward ssDNA. As previously reported, due to its high stability (T_m_ = 50.2 °C), the targeting of the complex **ON 1**:**cON** is very difficult and only a very faint band appears when **PNA 2** is used. The slow mobility band observed with **PNA 7** and **PNA 8** can again be attributed to the PNA:DNA duplex instead of the crosslinked product due to the smearing of the DNA band. On the contrary, targeting of the **ON 2**:**cON** complex proved to be facilitated by its reduced stability (T_m_ = 33.9 °C) and both **PNA 1** and **PNA 2** were shown to form an ICL. The introduction of the arginine side chain seems to be deleterious for both furan building blocks, while the introduction of the lysine modification gives conflicting effects, improving the ability of the building block **F** to form ICL, while inhibiting crosslink formation when using building block **f**. It is clear that the mere introduction of an additional positive charge in the backbone through building block modification does not guarantee a positive effect on duplex stability and crosslink capacity. In the case of **PNA 7**–**8**, the strand displacement experiments revealed a new band which can be attributed to the PNA:DNA duplex (Figure 4d) with charge differing for **PNA 7** and **8**, whereas for **PNA 9** only a smear was observed; in addition, a band of even lower mobility could be observed, which can be due to intermolecular cross-linking or to duplex-duplex interactions.

## 3. Materials and Methods

### 3.1. General

First, 2-((5-azidomethyl)uracil-1-yl)acetic acid [52] and 3-(2-furyl)propionic acid [47] were synthesized accordingly to previously reported procedures. The synthesis of **PNA 1**, **PNA 4**, and **PNA 7** was reported earlier [47].

All solvents and chemical reagents were purchased from Sigma-Aldrich (Overijse, Belgium), Acros (Geel, Belgium), Carlo Erba (Milano, Italy), TCI (Zwijndrecht, Belgium), Novabiochem (Overijse, Belgium), Link Technology (Bellshill, UK) in the highest purity available. Non-modified DNA sequences were purchased from Eurogentec (Seraing, Belgium).

^1^H-NMR and ^13^C-NMR spectra were recorded on a Bruker Avance 400 (Bruker, Brussels, Belgium) spectrometer operating at room temperature. Chemical shifts are reported in parts per million relative to the residual solvent peak. Multiplicities are reported as singlet (s), broad singlet (br s), doublet (d), doublet of doublets (dd), triplet (t), or multiplet (m).

LC-MS data were collected on an Agilent 1100 Agilent Technologies, Machelen, Belgium Series instrument with a Phenomenex Kinetex C18 100 Å column (150 × 4.6 mm, 5 μm at 35 °C) connected to an ESMSD type VL mass detector (quadrupole ion trap mass spectrometer, Agilent Technologies, Machelen, Belgium) with a flow rate of 1.5 mL/min was used with the following solvent systems: (A): 0.1% HCOOH in H_2_O and (B) MeCN. The column was flushed with 100% (A) for 2 min, then a gradient from 0 to 100% (B) over 6 min was used, followed by 2 min of flushing with 100% (B).

UPLC-ESI-Q data were collected on a Waters Acquity UPLC system (Waters, Sesto San Giovanni, Italy) equipped with a Waters Acquity UPLC BEH C18 column (50 × 2.1 mm, 1.7 µm) at 35 °C. A flow rate of 0.25 mL/min was used with the following solvent systems: (A): 0.2% formic acid (FA) in H_2_O and (B): 0.2% FA in MeCN. The column was flushed for 0.9 min with solvent (A), then a gradient from 0 to 50% (B) in 5.7 min. PNA purification was performed on a Agilent 1100 Series instrument equipped with a Phenomenex Jupiler C18 (5 µm, 300 Å, 250 × 10 mm) at 40 °C with a linear gradient from H_2_O 0.1% TFA to 50% MeCN 0.1% TFA in 30 min at a flow rate of 4.0 mL/min.

Concentrations of DNA and PNA solutions were measured with a Trinean DropSense96 UV-VIS droplet reader (Trinean, Gent, Belgium). Purification of the crosslinked samples was performed on an Agilent 1200b System (Agilent Technologies, Machelen, Belgium) equipped with a Waters X-bridge 130 Å Oligonucleotide C18 column (2.5 μM, 4.6 mm × 50 mm) at a column temperature of 50 °C. Acetonitrile and 0.1 M TEAA buffer with 5% acetonitrile were used as mobile phase and were applied through a gradient of 0–18% MeCN in 20 min.

### 3.2. Monomer Synthesis

*Synthesis of N-methyl-N-methoxy-Nα-Fmoc-Nω-Pbf-l-arginine-amide* (**1a**)*.* In a round-bottom flask equipped with a CaCl_2_ valve, Fmoc-l-Arg(Pbf)-OH (4.00 g, 6.2 mmol, 1 eq) was dissolved in 20 mL of dry DMF together with *N*,*N*,*N*′,*N*′-Tetramethyl-*O*-(1*H*-benzotriazol-1-yl)uronium hexafluorophosphate (HBTU, 3.52 g, 9.3 mmol, 1.5 eq). Subsequently, the solution was cooled to 0 °C with an ice bath, *N*,*N*-Diisopropylethylamine (DIPEA, 3.8 mL, 21.7 mmol, 3.5 eq) was added, and the mixture was allowed to react for 30 min. Next, *N*,*O*-dimethylhydroxylamine hydrochloride (1.21 g, 12.4 mmol, 2 eq) was added to the solution. The reagents were allowed to react for 10 min at 0 °C and 3 h 20 min at room temperature. Next, the reaction mixture was diluted with 200 mL EtOAc and washed with saturated KHSO_4_ (2 × 200 mL), saturated NaHCO_3_ (2 × 200 mL), and brine (200 mL). The organic phase was dried over Na_2_SO_4_ and the solvent was evaporated under reduced pressure. **1a** was obtained as a pale yellow foam (6.189 mmol, 99.8% yield). TLC (AcOEt) Rf: 0.29, MP (°C): 106.2–108.3 °C; ^1^H-NMR (CDCl_3_, 400 MHz) δ (ppm): 7.77 (d, *J* = 7.6 Hz, 2H), 7.59 (d, *J* = 6.7 Hz, 2H), 7.41 (t, *J* = 7.6 Hz, 2H), 7.31 (t, *J* = 6.6 Hz, 2H), 6.09 (3H, br s), 5.84 (d, *J* = 8.4 Hz, 1H), 4.80–4.65 (1H, m), 4.52–4.31 (1H, m), 4.20 (1H, t, *J* = 6.9 Hz), 3.74 (3H, s), 3.21 (3H, s), 2.94 (2H, s), 2.60 (3H, s), 2.53 (3H, s), 2.10 (3H, s), 1.84–1.54 (6H, m), 1.46 (6H, s); ^13^C-NMR (CDCl_3_, 100 MHz) δ (ppm): 172.1 (31), 158.8, 156.8, 156.0, 143.7, 141.3, 138.4, 132.9, 132.4, 127.8, 127.1, 125.1, 124.6, 120.0, 117.5, 86.4, 67.2, 61.7, 50.2, 47.1, 43.2, 41.0, 32.1, 30.8, 28.6, 24.7, 19.3, 17.9, 12.5; MS (ESI, MeOH): *m*/*z* calcd. for C_36_H_45_N_5_O_7_S [M]: 691.30397, found: 692.3 [M + H]^+^, 1383.5 [2M + H]^+^; HRMS (LTQ Orbitrap, MeOH) *m*/*z* found: 692.31076 [C_36_H_46_N_5_O_7_S]^+^; FT-IR (ATR) ν (cm^−1^): 3322.4 (w), 2952.0 (m), 2936.1 (m), 2356.7 (s), 2334.6 (s), 1700.0 (s), 1652.4 (s), 1615.5 (s), 1543.3 (s), 1448.5 (s), 1247.7 (m), 1104.6 (s), 1088.6 (s), 993.7 (s), 783.4 (s), 757.3 (s).

*Synthesis of N-methyl-N-methoxy-Nα-Fmoc-Nω-Boc-l-lysine-amide* (**1b**). In a round-bottom flask equipped with a CaCl_2_ valve, Fmoc-l-Lys(Boc)-OH (3.0 g, 6.4 mmol, 1 eq) was dissolved in 20 mL of dry DMF together with HBTU (3.64 g, 9.6 mmol, 1.5 eq). Subsequently, the solution was cooled to 0 °C with an ice bath, DIPEA (3.9 mL, 22.4 mmol, 3.5 eq) was added, and the mixture was allowed to react for 30 min. Next, *N*,*O*-dimethylhydroxylamine hydrochloride (1.25 g, 12.8 mmol, 2 eq) was added to the solution. The reagents were allowed to react for 10 min at 0 °C and 3 h 20 min at room temperature. Next, the reaction mixture was diluted with 200 mL EtOAc and washed with saturated KHSO_4_ (2 × 200 mL), saturated NaHCO_3_ (2 × 200 mL), and brine (200 mL). The organic phase was dried over Na_2_SO_4_ and the solvent was evaporated under reduced pressure. **1b** was obtained as a white foam in quantitative yield. TLC (AcOEt) Rf: 0.29, MP (°C): 50.2–50.5 °C; ^1^H-NMR (CDCl_3_, 400 MHz) δ (ppm): 7.78 (2H, d, *J* = 7.6 Hz), 7.63 (2H, t, *J* = 7.5 Hz), 7.42 (2H, t, *J* = 7.5 Hz), 7.34 (2H, tdd, *J* = 7.4 Hz, 2.9 Hz, 1.1 Hz), 5.55 (1H, d, *J* = 9.1 Hz), 4.81–4.72 (1H, br s), 4.65–4.53 (1H, s br), 4.39 (2H, d, *J* = 7.2 Hz), 4.24 (1H, t, *J* = 7.0 Hz), 3.80 (3H, s), 3.25 (3H, s), 3.14 (2H, t, *J* = 6.7), 1.86–1.70 (2H, m), 1.69–1.58 (1H, m), 1.56–1.49 (2H, m), 1.49–1.36 (10H, m); ^13^C-NMR (CDCl_3_, 100 MHz) δ (ppm): 172.6 (22), 156.2, 156.0, 144.0, 141.3, 127.7, 127.0, 125.1, 119.9, 79.1, 67.0, 61.6, 50.7, 47.2, 40.3, 32.5, 32.1, 29.6, 28.4, 22.5; MS (ESI, MeOH): *m*/*z* calcd. for C_28_H_37_N_3_O_6_ [M]: 511.26824, found: 512.2 [M + H]^+^, 1023.4 [2M + H]^+^ 534.2 [M + Na]^+^, 550.2 [M + K]^+^; HRMS (LTQ-Orbitrap, MeOH) *m*/*z* found: 534.25746 [C_28_H_37_N_3_O_6_Na]^+^; FT-IR (ATR) ν (cm^−1^): 3316.1 (w), 2972.7 (m), 2938.8 (m), 2863.8 (m), 2359.3 (s), 1700.0 (m), 1655.0 (s), 1513.1 (s), 1450.0 (s), 1390.9 (s), 1365.0 (s), 1246.2 (s), 1165.1 (s), 1040.4 (s), 990.9 (s), 860.9 (m), 758.7 (s), 738.9 (s).

*Synthesis of Nα-Fmoc-Nω-Pbf-l-argininal* (**2a**). In a three-neck flask connected with a nitrogen line through a bubble counter, **1a** (1.0 g, 1.44 mmol, 1 eq) was dissolved in 20 mL dry THF and cooled down to 0 °C with an ice bath. LiAlH_4_ (60.33 mg, 1.58 mmol, 1.1 eq) was dissolved in 1.5 mL dry THF and slowly added to the solution. The reaction was allowed to react for 30 min under stirring. Next, the reaction was quenched by addition of 20 mL saturated KHSO_4_. The organic fraction was evaporated under reduced pressure, after which the resulting water phase was diluted with 200 mL EtOAc and 200 mL saturated KHSO_4_. The organic phase was washed with saturated KHSO_4_ (2 × 100 mL) and brine (2 × 100 mL). The organic phase was dried with Na_2_SO_4_ and the solvent removed under reduced pressure. **2a** was obtained as a white foam (1.28 mmol, 88.6% yield). TLC (AcOEt) Rf: 0.56, MP (°C): 95.5–97.7 °C; ^1^H-NMR (CDCl_3_, 400 MHz) δ (ppm): 9.53 (1H, s), 7.77 (2H, d, *J* = 7.6 Hz), 7.60 (2H, d, *J* = 8.4 Hz), 7.41 (2H, t, *J* = 6.5 Hz), 7.32 (2H, m), 6.56 (3H, br s), 5.67 (1H, br s), 5.26 (1H, d, *J* = 9.9 Hz), 4.39 (2H, d, *J* = 7.2 Hz), 4.21 (1H, t, *J* = 7.2 Hz), 3.77–3.63 (1H, m), 3.19 (1H, m), 2.96 (2H, s), 2.57 (3H, s), 2.51 (3H, s), 2.11 (3H, s), 1.89–1.50 (6H, m), 1.47 (6H, s); ^13^C-NMR (CDCl_3_, 100 MHz) δ (ppm): 158.0, 155.9, 155.3, 144.4, 144.3, 141.2, 138.0, 134.3, 132.0, 128.1, 127.5, 125.8, 124.9, 120.6, 116.9, 86.8, 66.1, 55.4, 51.7, 47.1, 42.9, 28.8, 24.3, 19.4, 18.1, 12.7; MS (ESI, MeOH): *m*/*z* calcd. for C_34_H_40_N_4_O_6_S [M]: 632.26686, found: 633.3[M + H]^+^, 1265.6 [2M + H]^+^; HRMS (LTQ-Orbitrap, MeOH) *m*/*z* found: 633. [C_34_H_41_N_4_O_6_S]^+^; FT-IR (ATR) ν (cm^−1^): 3414.2 (m), 3341.4 (m), 2967.8 (m), 2936.1 (m), 1704.6 (s), 1684.8 (s), 1628.8 (m), 1576.9 (s), 1510.0 (s), 1448.5 (s), 1241.0 (m), 1086.9 (m), 990.2 (s), 869.7 (s), 852.4 (s), 818.9 (s), 758.8 (s), 738.1 (s).

*Synthesis of Nα-Fmoc-Nω-Boc-l-lysinal* (**2b**). In a three-neck flask connected with a nitrogen line through a bubble counter, **1b** (1.44 g, 2.8 mmol, 1 eq) was dissolved in 20 mL dry THF and cooled down to 0 °C with an ice bath. LiAlH_4_ (117.7 mg, 3.1 mmol, 1.1 eq) was dissolved in 1.5 mL dry THF and slowly added to the solution. The reaction was allowed to react for 32 min under stirring. Next, the reaction was quenched by addition of 20 mL saturated KHSO_4_. The organic fraction was evaporated under reduced pressure, after which the resulting water phase was diluted with 200 mL EtOAc and 200 mL saturated KHSO_4_. The organic phase was washed with saturated KHSO_4_ (2 × 100 mL), and brine (2 × 100 mL). The organic phase was dried with Na_2_SO_4_ and the solvent removed under reduced pressure. **2b** was obtained as a white foam (1.92 mmol, 68.7% yield). TLC (AcOEt) Rf: 0.56; ^1^H-NMR (CDCl_3_, 400 MHz) δ (ppm): 9.61 (1H, s), 7.80 (2H, d, *J* = 7.5Hz), 7.63 (2H, d, *J* = 7.4 Hz), 7.43 (2H, t, *J* = 7.3 Hz), 7.38–7.31 (2H, m), 4.80–4.55 (2H, m), 4.44 (2H, d, *J* = 7.0 Hz), 4.26 (1H, t, *J* = 6.9 Hz), 3.43–3.36 (2H, m), 3.15 (1H, br s), 1.91–1.37 (15H, m); ^13^C-NMR (CDCl_3_, 100 MHz) δ (ppm): 197.0, 156.5, 143.8, 141.3, 127.7, 127.1, 125.0, 120.0, 80.4, 66.9, 57.7, 49.7, 47.1, 42.8, 28.7, 28.4, 24.00; MS (ESI, MeOH): *m*/*z* calcd. for C_26_H_32_N_2_O_5_ [M]: 452.23112, found: 453.2 [M + H]^+^; 475.2 [M + Na]^+^, 491.1 [M + K]^+^; HRMS (LTQ-Orbitrap, MeOH) *m*/*z* found: 507.24656 [C_27_H_36_N_2_O_6_Na]^+^.

*Synthesis of Nα-Fmoc-Ψ(l-arginine-(Nω-Pbf))-glycine methyl ester* (**3a**). In a 50 mL round-bottom flask, **2a** (200 mg, 0.316 mmol, 1 eq) was dissolved in 10 mL dry MeOH, together with glycine methyl ester hydrochloride (79.4 mg, 0.632 mmol, 2 eq) and reacted for 15 min at 0 °C and 15 min at room temperature. Next, NaBH_3_CN (41.7 mg, 0.66 mmol, 2.1 eq) and acetic acid (36.9 μL, 0.66 mmol, 2.1 eq) were added. The pH of the solution was checked to be around 4.5. The reaction mixture was stirred overnight at room temperature. Next, the organic solvent was removed, and the crude was taken up with 20 mL EtOAc and washed with saturated NaHCO_3_ (2 × 20 mL) and brine (2 × 20 mL). The organic phase was dried over Na_2_SO_4_ and evaporated under reduced pressure. The product was purified using flash chromatography (DCM:MeOH 96:4 to DCM:MeOH 94:6), which yielded **3a** as a white foam (0.184 mmol, 58.3% yield). TLC (AcOEt) Rf: 0.28, MP (°C): 69.5–71.4 °C; ^1^H-NMR (CDCl_3_, 400 MHz) δ (ppm): 7.76 (2H, d, *J* = 7.0 Hz), 7.60 (2H, d, *J* = 8.0 Hz), 7.40 (2H, t, *J* = 7.2 Hz), 7.31 (2H, m), 6.13 (3H, s), 5.51 (1H, d, *J* = 7.3 Hz), 4.41 (2H, d, *J* = 4.4 Hz), 4.20 (1H, t, *J* = 6.7 Hz), 3.73 (3H, s), 3.70–3.62 (1H, m), 3.46 (1H, d, *J* = 17.6 Hz), 3.38 (1H, d, *J* = 16.7 Hz), 3.33–3.11 (2H, m), 2.94 (2H, s), 2.73–2.62 (2H, m), 2.60 (3H, s), 2.53 (3H, s), 2.09 (3H, s), 1.86 (1H, s), 1.70–1.47 (4H, m), 1.45 (6H, s); MS (ESI, MeOH): *m*/*z* calcd. for C_37_H_47_N_5_O_7_S [M]: 705.31962, found: 706.3 [M + H]^+^, 1411.7 [2M + H]^+^; HRMS (LTQ-Orbitrap, MeOH) *m*/*z* found: 706.3258 [C_37_H_48_N_5_O_7_S]^+^; FT-IR (ATR) ν (cm^−1^): 3325.6 (m), 2936.1 (m), 2917.2 (m), 1718.4 (m), 1700.8 (m), 1622.6 (m), 1560.7 (s), 1546.8 (s), 1448.8 (s), 1242.3 (m), 1153.9 (s), 1089.3 (s), 1033.6 (s), 993.1 (s), 851.7 (s), 807.7 (s), 781.3 (s), 760.1 (s), 739.4 (s).

*Synthesis of Nα-Fmoc-Ψ(l-lysine-(Nω-Boc))-glycine methyl ester* (**3b**). In a 50 mL round-bottom flask, **2b** (861 mg, 1.90 mmol, 1 eq) was dissolved in 10 mL dry MeOH, together with glycine methyl ester hydrochloride (477.8 mg, 3.81 mmol, 2 eq) and reacted for 15 min at 0 °C and 15 min at room temperature. Next, NaBH_3_CN (251.1 mg, 4.00 mmol, 2.1 eq) and acetic acid (228 μL, 4.00 mmol, 2.1 eq) were added. The pH of the solution was checked to be around 4.5. The reaction mixture was stirred overnight at room temperature. Next, the organic solvent was removed, and the crude was taken up with 20 mL EtOAc and washed with saturated NaHCO_3_ (2 × 20 mL) and brine (2 × 20 mL). The organic phase was dried over Na_2_SO_4_ and evaporated under reduced pressure. The crude was purified using flash chromatography (DCM to DCM:MeOH 96:4), which yielded **3b** as a white foam (1.66 mmol, 87.2% yield). TLC (AcOEt) Rf: 0.26; ^1^H-NMR (CDCl_3_, 400 MHz) δ (ppm): 7.79 (2H, d, *J* = 7.5 Hz), 7.63 (2H, d, *J* = 7.1 Hz), 7.42 (2H, t, *J* = 7.4 Hz), 7.34 (2H, t, *J* = 7.4 Hz), 5.13 (1H, d, *J* = 7.6 Hz), 4.61 (1H, br s), 4.43 (2H, d, *J* = 8.8 Hz), 4.24 (1H, t, *J* = 6.7 Hz), 3.78–3.63 (4H, m),3.49 (1H, d, *J* = 15.3 Hz), 3.40 (1H, d, *J* = 16.8 Hz), 3.19–3.03 (2H, m), 2.80–2.64 (2H, m), 1.86 (1H, br s), 1.59–1.30 (15H, m); ^13^C-NMR (CDCl_3_, 100 MHz) δ (ppm): 172.8, 156.4, 156.1, 144.0, 141.3, 127.7, 127.0, 125.1, 119.9, 79.1, 66.5, 53.4, 52.8, 51.8, 50.7, 47.4, 40.2, 32.6, 29.8, 28.4, 23.0; MS (ESI, MeOH): *m*/*z* calcd. for C_29_H_39_N_3_O_6_ [M]: 525.28389, found: 526.2 [M + H]^+^, 548.3 [M + Na]^+^, 1051.5 [2M + H]^+^; HRMS (LTQ-Orbitrap, MeOH) *m*/*z* found: 526.29116 [C_29_H_40_N_3_O_6_]^+^.

*Synthesis of Fmoc*-PNA*(5L-Arg(Pbf))-N_3_-OMe* (**4a**). In a round-bottom flask, 2-azidoacetic acid (63.6 μL, 0.85 mmol, 2 eq) and 3-hydroxy-1,2,3-benzotriazin-4 (3 h)-one (DhBtOH) (138.7 mg, 0.85 mmol, 2 eq) were dissolved in 5 mL of dry DMF and cooled to 0 °C. Then, 1-(3-dimethylaminopropyl)-3-ethylcarbodiimide hydrochloride (EDC·HCl) (162.9 mg, 0.85 mmol, 2 eq) and DIPEA (148.2 μL, 0.85 mmol, 2 eq) were added and stirred for 10 min at 0 °C, followed by 20 min at room temperature. Next, the reaction was cooled again to 0 °C and **3a** was added (300.5 mg, 0.425 mmol, 1 eq). The reaction was stirred for 5 min at 0 °C and for 5 h at room temperature. The reaction mixture was then diluted with EtOAc (100 mL) and washed with saturated KHSO_4_ (2 × 100 mL), saturated NaHCO_3_ (2 × 100 mL), and brine (100 mL). The organic phase was dried over Na_2_SO_4_ and the solvent was evaporated under reduced pressure. The crude was purified using flash chromatography (AcOEt:Hexane 8:2 to AcOEt) and yielded **4a** as a white foam (0.272 mmol, 63.9% yield). TLC (AcOEt) Rf: 0.28, MP (°C): 85.1–85.3; ^1^H-NMR (CDCl_3_, 400 MHz, main rotamer) δ (ppm): 7.77 (2H, d, *J* = 7.6 Hz), 7.59 (2H, d, *J* = 7.3 Hz), 7.40 (2H, t, *J* = 7.5 Hz), 7.31 (2H, m), 6.28 (3H, s), 5.62 (1H, d, *J* = 7.6 Hz), 4.43–4.30 (2H, m), 4.22–4.13 (1H, m), 4.06 (2H, s), 3.86 (2H, s), 3.82–3.77 (1H, m), 3.74 (3H, s), 3.44–3.12 (4H, m), 2.94 (2H, s), 2.60 (3H, s), 2.53 (3H, s), 2.10 (3H, s), 1.75–1.50 (4H, m), 1.45 (6H, s); ^13^C-NMR (CDCl_3_, 100 MHz, main rotamer) δ (ppm): 169.2, 168.6, 159.2, 157.0, 155.9, 143.8, 141.3, 138.7, 132.7, 127.8, 127.1, 125.1, 124.9, 120.0, 117.8, 114.7, 86.6, 66.8, 52.9, 52.4, 51.5, 50.4, 49.8, 47.2, 43.2, 41.0, 28.6, 25.2, 25.0, 19.3, 17.5, 12.5 (21); MS (ESI, MeOH): *m*/*z* calcd. for C_39_H_48_N_8_O_8_S [M]: 788.33158, found: 789.2 [M + H]^+^; HRMS (LTQ-Orbitrap, MeOH) *m*/*z* found: 789.33886 [C_39_H_49_N_8_O_8_S]^+^; FT-IR (ATR) ν (cm^−1^): 3449.1 (m), 3426.9 (m), 3340.3 (w), 2939.3 (w), 2104.1 (s), 1745.9 (s), 1714.5 (s), 1653.8 (s), 1616.2 (s), 1547.4 (s), 1450.1 (s), 1369.3 (s), 1241.1 (m), 1217.8 (m), 1154.4 (s), 1103.5 (s), 1088.7 (s), 992.2 (s), 808.4 (m), 758.5 (s), 740.0 (s).

*Synthesis of Fmoc*-PNA*(5L-Lys(Boc))-N_3_-OMe* (**4b**). In a round-bottomed flask, 2-azidoacetic acid (72.6 μL, 0.97 mmol, 2 eq) and DhBtOH (158.2 mg, 0.97 mmol, 2 eq) were dissolved in 5 mL of dry DMF and cooled to 0 °C. Then, EDC·HCl (185.9 mg, 0.97 mmol, 2 eq) and DIPEA (169.2 μL, 0.97 mmol, 2 eq) were added and stirred for 10 min at 0 °C, followed by 20 min at room temperature. Next, the reaction was cooled again to 0 °C and **3b** was added (255.0 mg, 0.48 mmol, 1 eq). The reaction was stirred for 5 min at 0 °C and for 3 h at room temperature. The reaction mixture was then diluted with EtOAc (100 mL) and washed with saturated KHSO_4_ (2 × 100 mL), saturated NaHCO_3_ (2 × 100 mL), and brine (100 mL). The organic phase was dried over Na_2_SO_4_ and the solvent was evaporated under reduced pressure. The crude was purified using flash chromatography (AcOEt:Hexane 6:4) and yielded **4b** as a pale yellow foam (0.360 mmol, 74.2% yield). TLC (AcOEt) Rf: 0.28, MP (°C): 51.3–51.6; ^1^H-NMR (CDCl_3_, 400 MHz, main rotamer) δ (ppm): 7.79 (2H, d, *J* = 7.4 Hz), 7.61 (2H, d, *J* = 7.2 Hz), 7.43 (2H, t, *J* = 7.3 Hz), 7.35 (2H, t, *J* = 7.3 Hz), 5.05 (1H, d, *J* = 5.7 Hz), 4.60 (1H, br s), 4.52–4.39 (2H, m), 4.22 (1H, d, *J* = 6.8 Hz), 4.01 (2H, s), 3.82 (2H, s), 3.78 (3H, s), 3.72–3.62 (1H, m), 3.29–3.18 (2H, m), 3.17–3.07 (2H, m), 1.58–1.37 (15H, m); ^13^C-NMR (CDCl_3_, 100 MHz, main rotamer) δ (ppm): 169.1, 168.4, 156.5, 156.1, 143.8, 141.3, 127.7, 127.1, 125.0, 120.0, 79.1, 66.6, 52.8, 52.4, 50.5, 49.9, 49.2, 47.3, 40.1, 32.4, 29.8, 28.4, 22.8; MS (ESI, MeOH): *m*/*z* calcd. for C_31_H_40_N_6_O_7_ [M]: 608.29585, found: 648.39 [M + K]^+^; HRMS (LTQ-Orbitrap, MeOH) *m*/*z* found: 631.28507 [C_31_H_40_N_6_O_7_Na]^+^; FT-IR (ATR) ν (cm^−1^): 3350.5 (w), 2941.5 (w), 2103.9 (s), 1747.8 (s), 1699.8 (m), 1662.1 (m), 1521.4 (s), 1450.4 (s), 1365.3 (s), 1247.5 (s), 1210.8 (s), 1168.4 (s), 1079.8 (s), 1022.9 (m), 759.2 (s), 739.4 (s).

*Synthesis of Fmoc*-PNA*(5L-Arg(Pbf))-F-OMe* (**4c**). In a round-bottomed flask, 3-(2-furyl)propionic acid (119.1 mg, 0.85 mmol, 2 eq) and DhBtOH (138.6 mg, 0.85 mmol, 2 eq) were dissolved in 5 mL of dry DMF and cooled to 0 °C. Then, EDC·HCl (162.9 mg, 0.85 mmol, 2 eq) and DIPEA (148.2 μL, 0.85 mmol, 2 eq) were added and stirred for 10 min at 0 °C, followed by 20 min at room temperature. Next, the reaction was cooled again to 0 °C and **3a** was added (300.0 mg, 0.43 mmol, 1 eq). The reaction was stirred for 5 min at 0 °C and for 3 h at room temperature. The reaction mixture was then diluted with EtOAc (100 mL) and washed with saturated KHSO_4_ (2 × 100 mL), saturated NaHCO_3_ (2 × 100 mL), and brine (100 mL). The organic phase was dried over Na_2_SO_4_ and the solvent was evaporated under reduced pressure. The crude was purified using flash chromatography (AcOEt:Hexane 8:2 to AcOEt) and yielded **4c** as a white solid (0.347 mmol, 81.6% yield). TLC (AcOEt) Rf: 0.28, MP (°C): 75.1–75.3; ^1^H-NMR (CDCl_3_, 400 MHz, major rotamer) δ (ppm): 7.76 (2H, d, *J* = 7.6 Hz), 7.59 (2H, d, *J* = 7.5 Hz), 7.39 (2H, t, *J* = 7.5 Hz), 7.32–7.21 (3H, m), 6.33–6.23 (4H, m), 6.02–5.96 (1H, m), 5.66 (1H, d, *J* = 7.4 Hz), 4.36 (2H, d, *J* = 7.3 Hz), 4.20–4.10 (1H, m), 4.06 (2H, s), 3.73 (3H, s), 3.68–3.58 (1H, m), 3.35–3.24 (2H, m), 3.21–3.09 (2H, m), 3.00–2.89 (4H, m), 2.60 (3H, s), 2.57–2.50 (5H, m, 36), 2.10 (3H, s), 1.70–1.48 (4H, m), 1.45 (6H, s); ^13^C-NMR (CDCl_3_, 100 MHz, major rotamer) δ (ppm): 173.9, 169.6, 159.0, 157.0, 155.9, 154.3, 143.8, 141.3, 141.1, 138.6, 132.6, 127.7, 127.1, 125.1, 124.7, 120.0, 117.6, 110.3, 105.4, 86.5, 66.7, 52.7, 52.2, 51.1, 50.4, 47.2, 43.2, 41.0, 31.4, 28.6, 25.6, 25.1, 23.5, 19.3, 18.0, 12.5; MS (ESI, MeOH): *m*/*z* calcd. for C_44_H_53_N_5_O_9_S [M]: 827.35640, found: 828.2 [M + H]^+^, 850.21 [M + Na]^+^; HRMS (LTQ-Orbitrap, MeOH) *m*/*z* found: 828.36368 [C_44_H_54_N_5_O_9_S]^+^; FT-IR (ATR) ν (cm^−1^): 3341.4 (m), 2933.0 (m), 1718.2 (m), 1623.7 (m), 1543.8 (m), 1449.6 (m), 1241.3 (m), 1212.4 (m), 1151.5 (m), 1087.6 (s), 1006.0 (s), 810.7 (s), 736.6 (s).

*Synthesis of Fmoc*-PNA*(5L-Lys(Boc))-F-OMe* (**4d**). In a round-bottom flask, 3-(2-furyl)propionic acid (135.9 mg, 0.97 mmol, 2 eq) and DhBtOH (158.2 mg, 0.97 mmol, 2 eq) were dissolved in 5 mL of dry DMF and cooled to 0 °C. Then, EDC·HCl (185.9 mg, 0.97 mmol, 2 eq) and DIPEA (169.2 μL, 0.97 mmol, 2 eq) were added and stirred for 10 min at 0 °C, followed by 20 min at room temperature. Next, the reaction was cooled again to 0 °C and **3b** was added (255.0 mg, 0.49 mmol, 1 eq). The reaction was stirred for 5 min at 0 °C and for 3 h at room temperature. The reaction mixture was then diluted with EtOAc (100 mL) and washed with saturated KHSO_4_ (2 × 100 mL), saturated NaHCO_3_ (2 × 100 mL), and brine (100 mL). The organic phase was dried over Na_2_SO_4_ and the solvent was evaporated under reduced pressure. The crude was purified using flash chromatography (AcOEt:Hexane 8:2) and yielded **4d** as a white solid (0.364 mmol, 75.0% yield). TLC (AcOEt) Rf: 0.39, MP (°C): 47.7–48.0; ^1^H-NMR (CDCl_3_, 400 MHz, major rotamer) δ (ppm): 7.78 (2H, d, *J* = 7.6 Hz), 7.61 (2H, d, *J* = 8.6 Hz), 7.42 (2H, td, *J* = 7.4 Hz, 2.8 Hz), 7.33 (2H, t, *J* = 7.4 Hz), 7.28 (1H), 6.26 (1H, dd, *J* = 4.4 Hz, 2.5 Hz), 6.00 (1H, dd, *J* = 12.1 Hz, 3.0 Hz), 5.22 (1H, d, *J* = 7.8 Hz), 4.60 (1H, br s), 4.41–4.34 (2H, m), 4.23–4.17 (1H, m), 4.07–3.95 (2H, m), 3.81–3.60 (4H, m), 3.53–3.30 (2H, m), 3.18–3.06 (2H, m), 3.02–2.92 (2H, m), 2.54 (2H, t, *J* = 7.6 Hz), 1.62–1.40 (15H, m); ^13^C-NMR (CDCl_3_, 100 MHz, major rotamer) δ (ppm): 173.4, 169.6, 156.6, 156.1, 154.6, 143.9, 141.3, 141.1, 127.7, 127.1, 125.3, 120.0, 110.4, 105.4, 79.1, 66.6, 53.0, 52.6, 52.2, 49.7, 47.3, 39.8, 32.5, 31.3, 29.8, 28.4, 23.6, 22.8; MS (ESI, MeOH): *m*/*z* calcd. for C_36_H_45_N_3_O_8_ [M]: 647.32067, found: 648.3 [M + H]^+^; HRMS (LTQ-Orbitrap, MeOH) *m*/*z* found: 670.30989 [C_36_H_45_N_3_O_8_Na]^+^; FT-IR (ATR) ν (cm^−1^): 3312.4 (w), 2977.3 (m), 2934.5 (m), 2857.0 (s), 1748.5 (s), 1701.2 (m), 1641.5 (m), 1705.5 (m), 1450.0 (m), 1365.1 (s), 1246.4 (s), 1210.6 (s), 1169.8 (s), 1076.9 (s), 1012.8 (s), 884.4 (s), 862.9 (m), 738.5 (s).

*Synthesis of Fmoc*-PNA*(5L-Arg(Pbf))-T(N_3_)-OMe* (**4e**). In a round-bottom flask, 2-((5-azidomethyl)uracil-1-yl)acetic acid (134.0 mg, 0.59 mmol, 1.1 eq) and DhBtOH (380 mg, 0.59 mmol, 1.1 eq) were dissolved in 2 mL of dry DMF and cooled to 0 °C. Then, EDC·HCl (72.1 mg, 0.59 mmol, 1.1 eq) and DIPEA (103.1 μL, 0.96 mmol, 1.1 eq) were added and stirred for 10 min at 0 °C, followed by 20 min at room temperature. Next, the reaction was cooled again to 0 °C and **3a** was added (380 mg, 0.48 mmol, 1 eq). The reaction was stirred for 5 min at 0 °C and overnight at room temperature. The reaction mixture was then diluted with EtOAc (100 mL) and washed with saturated KHSO_4_ (2 × 100 mL), saturated NaHCO_3_ (2 × 100 mL), and brine (100 mL). The organic phase was dried over Na_2_SO_4_ and the solvent was evaporated under reduced pressure. The crude was purified using flash chromatography (AcOEt) and **4e** was obtained as a yellowish solid (0.28 mmol, 74.7% yield). TLC (AcOEt) Rf: 0.18, ^1^H-NMR (400 MHz, DMSO-*d*_6_, major rotamer) δ 11.58 (1H, s), 7.89 (2H, d, *J* = 7.5 Hz), 7.67 (2H, t, *J* = 7.5 Hz), 7.60 (1H, d, *J* = 10.2 Hz), 7.41 (2H, t, *J* = 7.3 Hz), 7.37–7.24 (3H, m), 6.66 (1H, br s), 6.40 (1H, br s), 4.83 (1H, d, *J* = 16.6 Hz), 4.68 (1H, d, *J* = 16.7 Hz), 4.40–4.20 (3H, m), 4.03 (2H, s), 3.70 (2H, s), 3.61 (2H, s), 3.39–3.35 (1H, m), 3.04 (2H, s), 2.94 (2H, s), 2.48 (3H, s), 2.43 (3H, s), 2.00 (3H, s), 1.55–1.28 (10H, m); ^13^C-NMR (100 MHz, DMSO-*d*_6_) δ 169.7, 167.9, 163.9, 157.9, 156.5, 156.4, 151.1, 146.0, 144.5, 144.3, 144.2, 141.2, 138.8, 137.7, 134.7, 132.4, 131.9, 128.1, 127.5, 125.6, 124.8, 120.6, 117.7, 116.7, 107.8, 86.8, 65.7, 52.7, 52.2, 51.8, 50.0, 48.6, 48.6, 48.4, 47.3, 42.9, 28.7, 26.2, 19.4, 18.1, 12.7; MS (ESI, MeOH): *m*/*z* calcd. for C_44_H_52_N_10_O_10_S [M]: 912.35886, found: 913.7 [M + H]^+^, 935.7 [M + Na]^+^, 951.7 [M + K]^+^.

*Synthesis of Fmoc*-PNA*(5L-Lys(Boc))-T(N_3_)-OMe* (**4f**). In a round-bottom flask, 2-((5-azidomethyl)uracil-1-yl)acetic acid (215.0 mg, 0.96 mmol, 2 eq) and DhBtOH (155.8 mg, 0.96 mmol, 2 eq) were dissolved in 5 mL of dry DMF and cooled to 0 °C. Then, EDC·HCl (183.1 mg, 0.96 mmol, 2 eq) and DIPEA (166.6 μL, 0.96 mmol, 2 eq) were added and stirred for 10 min at 0 °C, followed by 20 min at room temperature. Next, the reaction was cooled again to 0 °C and **3b** was added (251.0 mg, 0.48 mmol, 1 eq). The reaction was stirred for 5 min at 0 °C and for 3 h at room temperature. The reaction mixture was then diluted with EtOAc (100 mL) and washed with saturated KHSO_4_ (2 × 100 mL), saturated NaHCO_3_ (2 × 100 mL), and brine (100 mL). The organic phase was dried over Na_2_SO_4_ and the solvent was evaporated under reduced pressure. The crude was purified using flash chromatography (AcOEt:Hexane 8:2 to AcOEt) and yielded **4f** as a pale yellow foam (0.339 mmol, 71.0% yield). TLC (AcOEt) Rf: 0.41, MP (°C): 74.1–74.4; ^1^H-NMR (CDCl_3_, 400 MHz, major rotamer) δ (ppm): 9.04 (1H, s), 7.78 (2H, d, *J* = 7.4 Hz), 7.62 (2H, d, *J* = 7.5 Hz), 7.41 (2H, t, *J* = 7.4 Hz), 7.33 (2H, m), 7.19 (1H, s), 5.11 (1H, d, 8.2 Hz), 4.81–4.58 (2H, m), 4.58–4.40 (3H, m), 4.36–4.29 (1H, m), 4.13–3.94 (4H, m), 3.81 (3H, s), 3.82–3.74 (1H, m), 3.64–3.27 (2H, m), 3.21–3.06 (2H, m), 1.59–1.32 (15 H, m); ^13^C-NMR (CDCl_3_, 100 MHz, major rotamer) δ (ppm): 169.5, 167.9, 162.7, 156.4, 150.5, 144.1, 143.8, 141.3, 127.8, 127.1, 125.1, 120.0, 109.4, 79.3, 66.5, 52.9, 52.4, 51.8, 50.4, 49.5, 47.9, 47.1, 40.0, 31.8, 29.7, 28.4, 22.9; MS (ESI, MeOH): *m*/*z* calcd. for C_36_H_44_N_8_O_9_ [M]: 732.32313, found: 733.4 [M + H]^+^; 755.4 [M + Na]^+^; 771.4 [M + K]^+^; HRMS (LTQ-Orbitrap, MeOH) *m*/*z* found: 755.31235 [C_36_H_44_N_8_O_9_Na]^+^; FT-IR (ATR) ν (cm^−1^): 3331.9 (m), 2974.1 (s), 2945.6 (s), 2929.8 (s), 2102.1 (s), 1672.4 (m), 1517.8 (m), 1449.5 (m), 1364.9 (s), 1245.0 (s), 1212.6 (s), 1167.3 (s), 1103.5 (s), 1078.4 (s), 891.7 (m), 863.9 (m), 833.4 (s), 758.6 (s), 740.4 (s).

*Synthesis of Fmoc*-PNA*(5L-Arg(Pbf))-N_3_-OH* (**5a**). Here, **4a** (214.3 mg, 0.27 mmol, 1 eq) was solubilized in 11 mL THF and cooled to 0 °C with an ice bath. Next, a solution of Ba(OH)_2_·8H_2_O (128.5 mg, 0.41 mmol, 1.5 eq) in 11 mL H_2_O was added and left to react for 30 min at 0 °C and a further 35 min at room temperature. The reaction was quenched with 1M HCl (0.95 mL, 0.95 mmol, 2.5 eq), the organic fraction was evaporated under reduced pressure, and the pH of the solution was adjusted to 3.5. The product was allowed to precipitate for 2 h at 4 °C. Then, **5a** was collected as white solid through Buchner filtration and washed with H_2_O (0.186 mmol, 68.6% yield). TLC (AcOEt) Rf: 0.17, MP (°C): 101.4–101.7; ^1^H-NMR (DMSO-*d*_6_, 400 MHz, major rotamer) δ (ppm): 8.69–8.50 (1H, br s), 7.89 (2H, d, *J* = 7.0 Hz), 7.66 (2H, d, *J* = 7.9 Hz), 7.41 (2H, t, *J* = 6.4 Hz), 7.33 (2H, m), 7.21 (1H, d, *J* = 8.3 Hz), 6.58–6.33 (3H, br s), 4.29 (2H, m), 4.21 (1H, m), 3.97 (1H, d, *J* = 17.4 Hz), 3.87 (1H, d, *J* = 17.6 Hz), 3.67 (1H, m), 3.61 (2H, s), 3.21 (2H, m), 3.02 (2H, s), 2.95 (2H, s), 2.49 (3H, s), 2.44 (3H, s), 2.00 (3H, s), 1.77 (2H, s), 1.40 (8H, s); ^13^C-NMR (DMSO-*d*_6_, 100 MHz, major rotamer) δ (ppm): 170.7, 168.5, 158.1, 156.6, 156.4,144.3, 141.2, 137.7, 134.7, 131.9, 128.1, 127.5, 125.7, 124.8, 120.6, 116.7, 86.8, 67.5, 51.8, 50.0, 49.6, 48.1, 47.2, 42.9, 40.4, 28.7, 25.6, 19.4, 18.1, 12.7; MS (ESI, MeOH): *m*/*z* calcd. for C_38_H_46_N_8_O_8_S [M]: 774.31593, found: 775.2 [M + H]^+^; HRMS (LTQ-Orbitrap, MeOH) *m*/*z* found: 773.30756 [C_38_H_45_N_8_O_8_S]^−^; FT-IR (ATR) ν (cm^−1^): 3339.2 (w), 2971.0 (m), 2929.8 (m), 2106.0 (s), 1704.0 (m), 1617.3 (m), 1547.0 (s), 1449.8 (s), 1419.3 (m), 1241.8 (m), 1153.5 (s), 1089.7 (s), 1033.7 (s), 993.3 (s), 969.0 (s), 814.1 (m), 782.7 (s), 760.0 (s), 739.6 (s).

*Synthesis of Fmoc*-PNA*(5L-Lys(Boc))-N_3_-OH* (**5b**). Here, **4b** (203.2 mg, 0.33 mmol, 1 eq) was solubilized in 15 mL THF and cooled to 0 °C with an ice bath. Next, a solution of Ba(OH)_2_·8H_2_O (157.8 mg, 0.50 mmol, 1.5 eq) in 15 mL H_2_O was added and left to react for 30 min at 0 °C and further 11 min at room temperature. The reaction was quenched with 1M HCl (1.2 mL, 1.2 mmol, 3.5 eq) and the organic fraction was evaporated under reduced pressure and the pH of the solution was adjusted to 3.5. The product was allowed to precipitate overnight at 4 °C. Then, **5b** was collected as beige solid through Buchner filtration and washed with H_2_O (0.209 mmol, 63.3% yield). TLC (AcOEt) Rf: 0.19, MP (°C): 62.7–63.0; ^1^H-NMR (DMSO-*d*_6_, 400 MHz, major rotamer) δ (ppm): 13.01–12.45 (1H, br s), 7.89 (2H, d, *J* = 7.5 Hz), 7.65 (2H, d, *J* = 7.7 Hz), 7.42 (2H, t, *J* = 7.4 Hz), 7.34 (2H, td, *J* = 7.4 Hz, 1.9 Hz), 7.19 (1H, d, *J* = 9.1 Hz), 6.76 (1H, s), 4.37–4.25 (2H, m), 4.23–4.15 (2H, m), 4.04 (1H, d, *J* = 17.2 Hz), 3.99 (1H, d, *J* = 38.3 Hz), 3.87 (1H, d, *J* = 17.3 Hz), 3.70–3.56 (1H, m), 3.28–2.97 (2H, m), 2.94–2.82 (2H, m), 1.46–1.12 (15H, m) ^13^C-NMR (DMSO-*d*_6_, 100 MHz, major rotamer) δ (ppm): 170.7, 168.5, 156.6, 156.0, 144.3, 141.2, 128.1, 127.5, 125.6, 120.6, 77.8, 65.8, 52.0, 49.9, 49.8, 48.1, 47.3, 39.8, 31.7, 28.8, 25.6, 23.3; MS (ESI, MeOH): *m*/*z* calcd. for C_30_H_38_N_6_O_7_ [M]: 594.28020, found: 595.4 [M + H]^+^; HRMS (LTQ-Orbitrap, MeOH) *m*/*z* found: 593.27182 [C_30_H_37_N_6_O_7_]^−^; FT-IR (ATR) ν (cm^−1^): 2926.7 (m), 2111.0 (s), 1714.3 (s), 1699.9 (s), 1683.7 (s), 1655.1 (s), 1648.3 (s), 1527.5 (s), 1506.7 (s), 1449.1 (s), 1419.7 (s), 1366.0 (s), 1244.9 (m), 1163.7 (m), 1102.7 (s), 1079.8 (s), 1020.7 (m), 949.7 (s), 799.2 (s), 759.0 (s), 737.4 (s).

*Synthesis of Fmoc*-PNA*(5L-Arg_(_Pbf))-F-OH* (**5c**). **4c** (286.8 mg, 0.35 mmol, 1 eq) was solubilized in 15 mL THF and cooled to 0 °C with an ice bath. Next, a solution of Ba(OH)_2_·8H_2_O (164.0 mg, 0.52 mmol, 1.5 eq) in 15 mL H_2_O was added and left to react for 30 min at 0 °C and further 46 min at room temperature. The reaction was quenched with 1M HCl (1.2 mL, 1.2 mmol, 3.5 eq), the organic fraction was evaporated under reduced pressure, and the pH of the solution was adjusted to 3.5. The product was allowed to precipitate for 2 h at 4 °C. Then, **5c** was collected as a white solid through Buchner filtration and washed with H_2_O (0.223 mmol, 64.4% yield). TLC (AcOEt) Rf: 0.20, MP (°C): 108.2–108.4; ^1^H-NMR (DMSO-*d*_6_, 400 MHz, major rotamer) δ (ppm): 8.81–8.59 (1H, br s), 7.88 (2H, d, *J* = 7.5 Hz), 7.68 (2H, d, *J* = 6.8 Hz), 7.47 (1H, d, *J* = 10.6 Hz), 7.43–7.36 (2H, m), 7.33–7.24 (2H, m), 7.14 (1H, d, *J* = 9.5 Hz), 6.60–6.40 (3H, br s), 6.34–6.27 (1H, m), 6.13–6.03 (1H, m), 4.35–4.24 (2H, m), 4.23–4.13 (1H, m), 4.01 (1H, d, *J* = 16.8 Hz), 3.83 (1H, d, *J* = 17.8 Hz), 3.70–3.54 (3H, m), 3.33–3.21 (2H, m), 3.08–2.96 (2H, m), 2.94 (2H, s), 2.83–2.71 (2H, m), 2.47–2.51 (3H, s) 2.43 (3H, s), 2.00 (3H, s), 1.80–1.73 (2H, m), 1.39 (6H, s), 1.37–1.20 (2H, m); ^13^C-NMR (DMSO-*d*_6_, 100 MHz, major rotamer) δ (ppm): 172.3, 171.8, 157.9, 156.6, 156.4, 155.3, 144.3, 141.6, 141.2, 137.7, 134.8, 131.9, 128.1, 127.5, 125.6, 124.8, 120.6, 116.7, 110.8, 105.5, 86.7, 67.5, 52.4, 49.9, 48.2, 47.3, 42.9, 40.4, 31.1, 28.7, 25.6, 23.5, 19.4, 18.1, 12.7; MS (ESI, MeOH): *m*/*z* calcd. for C_43_H_51_N_5_O_9_S [M]: 813.34075, found: 814.2 [M + H]^+^, 836.1 [M + Na]^+^; HRMS (LTQ-Orbitrap, MeOH) *m*/*z* found: 814.34803 [C_43_H_52_N_5_O_9_S]^+^; FT-IR (ATR) ν (cm^−1^): 3329.0 (w), 2967.8 (m), 2936.0 (m), 1715.1 (s), 1617.1 (m), 1545.7 (m), 1449.0 (m), 1406.0 (s), 1242.9 (m), 1151.9 (m), 1088.5 (m), 1012.4 (s), 904.6 (s), 851.4 (s), 810.7 (s), 782.6 (s), 760.6 (s), 733.9 (s).

*Synthesis of Fmoc*-PNA*(5L-Lys(Boc)-F-OH* (**5d**). Here, **4d** (219.6 mg, 0.34 mmol, 1 eq) was solubilized in 15 mL THF and cooled to 0 °C with an ice bath. Next, a solution of Ba(OH)_2_·8H_2_O (160.4 mg, 0.51 mmol, 1.5 eq) in 15 mL H_2_O was added and left to react for 30 min at 0 °C and further 46 min at room temperature. The reaction was quenched with 1M HCl (1.2 mL, 1.2 mmol, 3.5 eq) and the organic fraction was evaporated under reduced pressure and the pH of the solution was adjusted to 3.5. The product was allowed to precipitate overnight at 4 °C. Then, **5d** was collected as beige solid through Buchner filtration and washed with H_2_O (0.142 mmol, 42% yield). TLC (AcOEt) Rf: 0.19; MP (°C): 51.4–51.8; ^1^H-NMR (DMSO-*d*_6_, 400 MHz, major rotamer) δ (ppm): 8.17–8.12 (1H, br s), 7.88 (2H, d, *J* = 7.4 Hz), 7.65 (2H, d, *J* = 7.1 Hz), 7.52–7.36 (3H, m), 7.36–7.25 (2H, m), 7.21 (1H, d, *J* = 9.2 Hz), 6.75 (1H, s), 6.36–6.25 (1H, m), 6.10 (1H, d, *J* = 2.6 Hz), 4.28 (2H, m), 4.18 (1H, m), 4.12–3.78 (2H, m), 3.74–3.62 (1H, s), 3.42–3.21 (2H, m), 2.87 (2H, s), 2.75 (2H, m), 2.58–2.46 (2H, m), 1.36 (15H, m); ^13^C-NMR (DMSO-*d*_6_, 100 MHz, major rotamer) δ (ppm): 171.9, 171.2, 156.5, 156.0, 155.2, 144.3, 141.6, 141.2, 128.0, 127.5, 125.5, 120.5, 110.8, 105.6, 77.8, 66.7, 52.6, 50.2, 48.1, 47.3, 40.4, 31.6, 30.7, 29.7, 28.7, 23.6, 23.3; MS (ESI, MeOH): *m*/*z* calcd. for C_35_H_43_N_3_O_8_ [M]: 633.30502, found: 634.4 [M + H]^+^; 672.3 [M + K]^+^; HRMS (LTQ-Orbitrap, MeOH) *m*/*z* found: 656.29424 [C_35_H_43_N_3_O_8_Na]^+^; FT-IR (ATR) ν (cm^−1^): 3314.8 (w), 2937.2 (m), 2357.5 (s), 2344.2 (s), 1701.0 (m), 1636.7 (m), 1507.6 (m), 1449.8 (s), 1365.9 (s), 1246.6 (m), 1166.2 (m), 1077.1 (s), 1014.1 (s), 859.5 (m), 759.6 (s), 736.3 (s).

*Synthesis of Fmoc*-PNA*(5L-Arg(Pbf))-T(N_3_)-OH* (**5e**). Here, **4c** (256.6 mg, 0.28 mmol, 1 eq) was solubilized in 10 mL THF and cooled to 0 °C with an ice bath. Next, a solution of Ba(OH)_2_·8H_2_O (133.0 mg, 0.42 mmol, 1.5 eq) in 15 mL H_2_O was added and left to react for 30 min at 0 °C and then a further 46 min at room temperature. The reaction was quenched with 1M HCl (0.84 mL, 0.84 mmol, 3.5 eq) and the organic fraction was evaporated under reduced pressure and the pH of the solution was adjusted to 3.5. The product was allowed to precipitate for 2 h at 4 °C. Then, **5c** was collected as white solid through Buchner filtration and washed with H_2_O (0.271 mmol, 96.3% yield). TLC (EtOAc) Rf: 0.1; ^1^H-NMR (400 MHz, DMSO-*d*_6_, major rotamer) δ 11.53 (1H, s), 7.89 (2H, d, *J* = 7.4 Hz), 7.67 (2H, t, *J* = 8.3 Hz), 7.60 (1H, d, *J* = 6.9 Hz), 7.41 (2H, t, *J* = 7.4 Hz), 7.40–7.25 (3H, m), 7.06 (1H, 1 br s), 6.82 (1H, br s), 6.54 (1H, br s), 4.52 (1H, s), 4.44–4.28 (2H, m), 4.22 (1H, d, *J* = 5.9 Hz), 4.03 (2H, s), 3.70–3.53 (3H, m), 3.04 (2H, s), 2.94 (2H, s), 2.49 (3H, s), 2.42 (3H, s), 2.00 (3H, s), 1.55–1.20 (10H, m); ^13^C-NMR (100 MHz, DMSO-*d*_6_) δ 171.4, 168.0, 163.9, 157.9, 156.5, 156.3, 151.1, 146.4, 144.3, 141.2, 137.7, 134.7, 131.9, 130.1, 129.4, 129.0, 128.0, 127.5, 125.7, 124.8, 120.5, 116.7, 107.6, 86.7, 65.6, 51.7, 49.9, 49.4, 48.6, 47.3, 42.9, 28.7, 25.6, 19.4, 18.1, 12.7; MS (ESI, MeOH): *m*/*z* calcd. for C_43_H_50_N_10_O_10_S [M]: 898.34321, found: 897.7 [M − H]^−^, 935.7 [M + Na]^+^, 951.7 [M + K]^+^; HRMS (LTQ-Orbitrap, MeOH) *m*/*z* found: 897.3360 [C_43_H_49_N_10_O_10_S]^−^.

*Synthesis of Fmoc*-PNA*(5L-Lys(Boc))-T(N_3_)-OH* (**5f**). Here, **4f** (232.8 mg, 0.32 mmol, 1 eq) was solubilized in 14 mL THF and cooled to 0 °C with an ice bath. Next, a solution of Ba(OH)_2_·8H_2_O (151.1 mg, 0.46 mmol, 1.5 eq) in 14 mL H_2_O was added and left to react for 30 min at 0 °C and a further 10 min at room temperature. The reaction was quenched with 1M HCl (1.1 mL, 1.1 mmol, 3.5 eq) and the organic fraction was evaporated under reduced pressure and the pH of the solution was adjusted to 3.5. The product was allowed to precipitate overnight at 4 °C. **5f** was then collected as white solid through Buchner filtration and washed with H_2_O (0.244 mmol, 77% yield). TLC (AcOEt) Rf: 0.21; MP (°C): 97.4–97.7; ^1^H-NMR (DMSO-*d*_6_, 400M Hz, major rotamer) δ (ppm): 11.57 (1H, s), 8.10 (1H, m), 7.90 (2H, d, *J* = 7.5 Hz), 7.69 (2H, d, *J* = 7.0 Hz), 7.60 (1H, s), 7.42 (2H, t, *J* = 7.4 Hz), 7.33 (2H, t, *J* = 7.4 Hz), 7.27 (1H, d, *J* = 8.7 Hz), 6.81–6.72 (1H, m), 4.74 (2H, dd, *J* = 57.4 Hz, 16.7 Hz), 4.40–4.28 (2H, m), 4.26–4.19 (1H, m), 4.13 (1H, d, *J* = 7.7 Hz), 4.17–3.87 (4H, m), 3.76–3.64 (1H, m), 3.63–3.50 (1H, m), 3.01–2.82 (2H, m), 1.56–1.14 (15H, m); ^13^C-NMR (DMSO-*d*_6_, 100 MHz, major rotamer) δ (ppm): 170.6, 167.7, 163.9, 156.6, 156.0, 151.1, 146.1, 144.4, 141.2, 128.1, 127.5, 125.7, 120.6, 107.7, 77.8, 65.8, 51.9, 51.5, 50.3, 48.6, 47.3, 47.2, 40.4, 32.0, 29.8, 28.7, 23.3; MS (ESI, MeOH): *m*/*z* calcd. for C_35_H_42_N_8_O_9_ [M]: 718.30747, found: 719.4 [M + H]^+^; 741.4 [M + Na]^+^; 757.4 [M + K]^+^; HRMS (LTQ-Orbitrap, MeOH) *m*/*z* found: 741.29822 [C_35_H_42_N_8_O_9_Na]^+^; FT-IR (ATR) ν (cm^−1^): 3317.9 (m), 2932.8 (m), 2100.8 (m), 1669.5 (m), 1517.9 (m), 1450.4 (m), 1389.0 (s), 1365.3 (s), 1338.8 (s), 1246.0 (m), 1165.9 (m), 1104.4 (s), 1080.1 (s), 948.7 (m), 864.4 (m), 760.2 (s), 740.4 (s).

### 3.3. *PNA* Synthesis

The synthesis of the PNAs was performed using a standard Fmoc-based manual synthesis protocol using **5a**, **5b**, **5c**, **5d**, **5e** and **5f** in addition to standard monomers, on a Rink amide ChemMatrix resin loaded with Fmoc-Gly-OH as first monomer (0.2 mmol/g), using HBTU/DIPEA as an activating mixture. Cleavage of the resin was performed using a TFA/m-cresol 9:1 solution for all the PNA strands except for **PNA 2** and **PNA 3**, which were cleaved with a TFA/m-cresol/thioanisole 8:1:1 solution. The cleavage step was carried out for 1 h, twice.

General protocol for click reaction: different solutions were prepared: 200 mM solution of 3-(furan-2-yl)-*N*-(prop-2-yn-1-yl)propanamide in MeOH, 200 mM solution of copper sulfate in H_2_O, and 200 mM solution of sodium ascorbate in H_2_O. Reaction was carried out with a final PNA concentration (from crude PNA) of 2 mM using a molar ratio alkyne/ascorbate/Cu(II) of 2:4:2. The mixture was then left to react for 3 h before the purification.

General protocol for DA and retro-DA: for a 5 μmol scale of resin-loaded PNA in a 500 μL Eppendorf tube, 200 μL of a 0.25 M solution of *N*-(*N*-Boc-2-amminoethyl)maleimide in DMF were added and the mixture was heated at 90 °C for 7 h. The resin beads were then transferred to an empty SPE tube equipped with PE frit, and washed carefully with DMF and DCM. The resin was then submitted to a cleavage step and the resulting crude was solubilized in 5 mL milliQ (1 mM solution, calculated on the crude PNA) and heated at 90 °C for 5 h.

**PNA 2** (HPLC-HRMS): Rt: 15.58; MW: 3158.2976; *m*/*z* found: 1054.1077 [MH_3_]^3+^, 790.8324 [MH_4_]^4+^, 632.8675 [MH_5_]^5+^; **PNA 3** (HPLC-HRMS): Rt: 15.93; MW: 3186.3051; *m*/*z* found: 1063.4428 [MH_3_]^3+^, 797.8342 [MH_4_]^4+^, 638.4686 [MH_5_]^5+^, 532.2249 [MH_6_]^6+^; **PNA 5** (HPLC-HRMS): Rt: 35.70; MW: 3296.3502; *m*/*z* found: 1100.1247 [MH_3_]^3+^, 825.3451 [MH_4_]^4+^, 660.4780 [MH_5_]^5+^, 550.5658 [MH_6_]^6+^; **PNA 6** (HPLC-HRMS): Rt: 16.03; MW: 3324.3570; *m*/*z* found: 1109.4605 [MH_3_]^3+^, 832.3470 [MH_4_]^4+^, 666.0793 [MH_5_]^5+^, 555.2337 [MH_6_]^6+^; **PNA 8** (HPLC-HRMS): Rt: 15.60; MW: 3420.3795; *m*/*z* found: 1141.4685 [MH_3_]^3+^, 856.3526 [MH_4_]^4+^, 685.2841 [MH_5_]^5+^, 571.2374 [MH_6_]^6+^; **PNA 9** (HPLC-HRMS): Rt: 15.77; MW: 3448.3870; *m*/*z* found: 1150.8037 [MH_3_]^3+^, 863.3548 [MH_4_]^4+^, 690.8850 [MH_5_]^5+^, 575.9053 [MH_6_]^6+^.

### 3.4. Melting Temperature Measurements

Thermal denaturation profiles were measured by monitoring the absorbance at 260 nm from 18 °C to 90 °C and from 90 °C to 18 °C with a heating rate of 1 °C/min and recording every 0.1 °C (3 cycles). Measurement conditions: strand concentration = 5 µM in pH 7.0 PBS buffer (100 mM NaCl, 10 mM NaH_2_PO_4_). Melting curves were recorded on a Varian Cary 300 Bio instrument equipped with a six-cell thermostated cell holder, and the melting temperatures were calculated from the first derivative of the heating/cooling curves using the Cary 300 Bio software (v 3.00 (182), Varian, Machelen, Belgium).

### 3.5. Crosslinking Experiments

All experiments were conducted in a total volume of 50 μL, at 10 μM strand concentration in pH 7.0 PBS buffer (100 mM NaCl, 10 mM phosphates) and the complexes were allowed to form by slow annealing from 90 °C to room temperature. Crosslink experiments were performed by adding a freshly prepared stock solution of *N*-Bromosuccinimide (NBS, 0.5 nmol/2 µL) and to start the reaction, 1 equivalent (=0.5 nmol) of NBS was added. This was repeated every 15 min until four equivalents of NBS were added. Temperature was kept constant in an Eppendorf thermomixer comfort at 25 °C.

For strand displacement experiments, the DNA duplex was first allowed to anneal before the addition of the PNA strand, and the solution was left to equilibrate for 14 h at 37 °C before running the crosslink experiment at the same temperature.

### 3.6. Polyacrylamide Gel-Electrophoresis

Crosslink samples were analyzed on a 20% polyacrylamide gel (acrylamide:bisacrylamide 19:1) prepared in 1× Tris-Borat-EDTA (TBE) buffer containing 7 M urea. The temperature of the gel was stabilized with a Julabo F12 at 25 °C. The power supply used for gel electrophoresis was a consort EV202 and a constant voltage of 230 V was used to run the gels. Gels were stained with SYBR gold (Thermo Fisher Scientific, Life Technologies, Merelbeke, Belgium) and pictures were taken with an Autochemi imaging system (UVP). 4 µL of the crosslink solution (10 µM) were mixed with 16 µL formamide and from this mixture 8 µL were loaded on the gel.

### 3.7. *RP-HPLC* Purification and *MALDI-TOF* Analysis of Crosslinked Products

Prior to MALDI analysis, each crosslinked product was purified using reverse phase HPLC (RP-HPLC). Purification was performed on an Agilent 1200b System equipped with a Waters X-bridge 130 Å Oligonucleotide C18 column (2.5 μM, 4.6 mm × 50 mm) at a column temperature of 50 °C. Acetonitrile and 0.1 M TEAA buffer with 5% acetonitrile were used as mobile phase and were applied through a gradient of 0–18% MeCN in 20 min. The masses of the PNA-DNA crosslinked samples were analyzed by MALDI-TOF on an ABI Voyager DE-STR system equipped with a high performance nitrogen laser (337 nm). PNA–DNA crosslinked samples were analyzed in linear, positive mode and samples were prepared using 2,5-dihydroxybenzoic acid (15 mg in 100 μL milliQ and 50 μL MeCN with 1% TFA) as matrix.

## 4. Conclusions

The present results show that the synthesis of multifunctional PNA molecules with a cationic substituent on the backbone and furan moieties instead of or on the nucleobase can be achieved by careful choice of reaction conditions, with a Diels–Alder reaction being a part of the chemical toolbox for temporary protection of the furan moieties. The crosslink properties of furan–PNA strands containing modified PNA building blocks featuring a lysine or arginine side chain in addition to the furan moiety were evaluated towards single stranded DNA sequences. The results show that the crosslink event could be enhanced and optimized by varying the furan building block and side chain modification. Overall, it was seen that the introduction of a lysine side chain in the furan-containing building block resulted in a higher crosslink yield compared to non-modified or arginine-modified PNA. This observation can be attributed to pre-organization of the PNA probe and a favorable electrostatic interaction with the negatively charged DNA-backbone. Moreover, it cannot be excluded that the lysine side chain helps in the crosslinking process. In addition, a correlation was seen between the used furan building block and the preferred nucleobases for crosslinking. While crosslinking towards adenine was favored with furan building block **F**, building block **f** is preferred cytosine crosslinking. These results indicate that the crosslinking event can be fine-tuned for each individual target sequence. Through the current screening, it has been further possible to define a suitable doubly modified PNA building block containing a furan moiety as well as a positively charged lysine side chain that allows enhancing cross-link formation of a PNA strand towards a complementary ssDNA strand in a strand invasion scenario.

On the other hand, the positioning of the furan on the uracil base (**T**(**f**) series) generates a PNA monomer still able to give Watson–Crick type of recognition with high sequence selectivity, which can compete with DNA:DNA formation, but is not able to cross-link with the DNA, thus leaving the activated furan moiety in the major groove. Though not useful for ICL, this reactive species could be profitably used for the reaction of the PNA:DNA duplex with other groups, e.g., with proteins or small molecules for labeling.

The results obtained in this study demonstrate the possibility to improve probe reactivity by introduction of functional modifications such as positively charged side chains. It is however clear that considerable effort is still needed in order to optimize the reaction yield and identifying the optimal analytical tool that will help in a fast and reliable quantification of the ICL yield. Studies focusing on the improvement of the efficiency of the crosslink formation by variation of the geometry of the furan monomers and their placement along the strand are currently underway. The ICL extent obtained in the current context was superior to that previously obtained with uncharged PNAs, but still far from the quantitative reaction which would be needed for the blockage of aberrant genes in therapy. For analytical assays however, even a slight increase in ICL yield can be highly beneficial.

## Figures and Tables

**Table 1:** Melting temperatures (T_m_) for PNA:DNA, measured by UV absorbance at 260 nm in PBS buffer at pH 7. Strand concentration: 5 μM. PNA sequence: Ac-GGG CA**X** GAT CT-Gly-NH_2_, where **X** stands for the modified monomer (**O**, **K**, **R**, **F**, **f**, **T** (**f**) see Figure 1 for structural details), DNA sequence: 5′-AGA TCN TGC CC-3′, where N=A (**ON 1**), C (**ON 2**), G (**ON 3**) or T (**ON 4**). Numbers inside parentheses indicate the T_m_ difference with respect to T_m_s obtained for the sequences with unmodified backbone (**PNA 1**, **PNA 4** and **PNA 7**). Data for **PNA ref** (Ac-GGGCATGATCT-Gly-NH_2_) are from [47]. Light grey lines indicate PNAs bearing a lysine side chain modification, dark grey lines indicates PNAs bearing an arginine side chain modification.

	ON 1, N=A	ON 2, N=C	ON 3, N=G	ON 4, N=T
**PNA 1**, X=O, **F**	47.4	47.2	48.0	47.2
**PNA 2**, X=K (∆T_m_), **F**	47.3 (−0.1)	50.4 (+3.2)	50.7 (+2.7)	50.4 (+3.2)
**PNA 3**, X=R (∆T_m_), **F**	45.7 (−1.3)	48.6 (+1.4)	43.7 (−4.3)	48.7 (+1.3)
**PNA 4**, X=O, **f**	50.0	49.8	59.5	51.2
**PNA 5**, X=K (∆T_m_), **f**	50.3 (+0.3)	50.8 (+1.0)	62.3 (+2.8)	54.8 (+3.6)
**PNA 6**, X=R (∆T_m_), **f**	51.4 (+1.4)	50.9 (+1.1)	63.4 (+3.9)	55.0 (+3.8)
**PNA 7**, X=O, **T**(**f**)	69.0	51.0	55.8	57.8
**PNA 8**, X=K (∆T_m_), **T**(**f**)	71.0 (+2.0)	54.0 (+3.0)	59.6 (+3.8)	59.4 (+1.6)
**PNA 9**, X=R (∆T_m_), **T**(**f**)	71.0 (+2.0)	54.5 (+3.5)	59.7 (+3.9)	58.3 (+0.5)
**PNA ref**, X=O, **T**	68.9	50.0	55.4	56.8

## Supplementary Materials

The following are available online: solid phase Diels–Alder optimization; HPLC-DAD-HRMS chromatogram of synthesized PNAs; full PAGE images; MALDI analysis of the XL products; and the mechanism of ICL formation.
