# Supplementary material for: Synthesis and Improved Cross-Linking Properties of C5-Modified Furan Bearing PNAs
Source: Molecules. 2017 Nov 20;22(11):2010. doi: 10.3390/molecules22112010 (PMC6150320; doi:10.3390/molecules22112010)
Supplement: Supplementary file 1 [file molecules-22-02010-s001.pdf]

molecules

Synthesis and improved cross-linking properties of C5-modified furan bearing PNAs

Joke Elskens[1], Alex Manicardi[1,2], Valentina Costi[2], Annemieke Madder[1]*, Roberto Corradini[2]*

[1] Organic and Biomimetic Chemistry Research Group, Department of Organic and Macromolecular Chemistry, Ghent University, Krijgslaan 281-S4, Gent 9000, Belgium.
[2] Department of Chemistry, Life Sciences and Environmental Sustainability, University of Parma, Parco Area delle Scienze 17/A, Parma 43124, Italy

Supplementary Material - Table of contents

Solid Phase Diels-Alder optimization **2-5**
Solvent and maleimide concentration optimization 2
Figure S1: UPLC analysis of products obtained using N-(N-Boc-2-amminoethyl)maleimide as protecting group 3
Maleimide concentration and equivalents optimization 3
Figure S2: relative by-product formation 4
Maleimide equivalents and reaction time optimization 4
Figure S3: relative by-product formation 5
Visualization of the retro-DA reaction time course 6
Figure S4: time course of the retro-DA reaction 6
HPLC-DAD-HRMS chromatograms **7**
Figure S5: Overview of the PNA sequences and furan-PNA building blocks 7
Figure S6: HPLC-DAD-HRMS chromatogram of purified **PNA 2** 8
Figure S7: HPLC-DAD-HRMS chromatogram of purified **PNA 3** 9
Figure S8: HPLC-DAD-HRMS chromatogram of purified **PNA 5** 10
Figure S9: HPLC-DAD-HRMS chromatogram of purified **PNA 6** 11
Figure S10: HPLC-DAD-HRMS chromatogram of purified **PNA 8** 12
Figure S11: HPLC-DAD-HRMS chromatogram of purified **PNA 9** 13
PAGE analysis **14**
Figure S12: PAGE analysis of the cross-link experiment between **ON1-ON4** and **PNA1-PNA3** 14
Figure S13: PAGE analysis of the cross-link experiment between **ON1-ON4** and **PNA4-PNA6** 15
Figure S14: PAGE analysis of the cross-link experiment between **ON1-ON4** and **PNA7-PNA9** 15
Figure S15: PAGE analysis of the strand displacement experiment between **ON1-ON2** and **PNA1-PNA9** 16
MALDI analysis **17**
Figure S16: MALDI analysis of the cross-link product of **ON2** and **PNA4** 17
Figure S17: HPLC trace and MALDI analysis of the cross-link product of **ON1** and **PNA2** 18
Mechanism of Interstrand Crosslink formation **19**
Figure S18: Schematic representation of interstrand crosslink formation 19

Solid Phase Diels-Alder optimization

In order to optimize the Diels-Alder reaction conditions on the solid phase, a small PNA sequence was N-capped with 2-furanpropionic acid (Fur-GATCT-Gly-NH_2, loading 7,45 mg/µmol).

Solvent and maleimide concentration optimization

<u>General protocol</u>

1- Load a 200 µL Eppendorf with 0.2 µmol resin (approx. 1.86 mg);

2- Prepare desired solutions of N-(N-Boc-2-amminoethyl)maleimide, and transfer them to the Eppendorf tube;

3- Allow Diels-Alder reaction for 15 hours at 90°C

4- Transfer the resin beads in a 1 mL SPE tube equipped with PE frit and wash the resin with DMF/DCM;

5- Add 100 μL of a 10% m-cresol solution in TFA and allow to react for 1h30';

6- Percolate the solution in a 2 mL Eppendorf tube, and wash the resin with 80 μL TFA;

7- Add 1.8 mL ethyl ether and allow to precipitate for 2h at -20 °C;

8- Recover the precipitate by centrifugation, and wash the pellet with ethyl ether;

9- Redissolve the dry pellet in 500 μL mQ, transfer in a 500 μL Eppendorf tube and heat for 15 hours at 90 °C;

10- Submit the samples to UPLC-MS analysis.

Solution used

Eq. maleimide / Concentration	Solvent: Toluene	Solvent: DMF
100 eq / 2 M	Test A1	Test B1
50 eq / 1 M	Test A2	Test B2
20 eq / 0.5 M	Test A3	Test B3
10 eq / 0.25 M	Test A4	Test B4
5 eq / 0.125 M	\	Test B5

Target identification

PNA \ Charge	No charge	2+	3+	4+
Target	1546.5	774	516	387
Alkylated	1712.7	857	571	429
Cycloaddition by-prod	1686.6	844	563	422

Results: when toluene is used as a solvent, it is not possible to prevent furan alkylation or the formation of a by-product with an additional unit of maleimide connected to the PNA strand. When using DMF it is possible to prevent the alkylation (20 eq / 0.5 M seems enough) but not the formation of the extra by-product. In all cases the alkylation of the furan is inversely proportional to the amount of maleimide used, while the formation of the by-product is directly proportional.

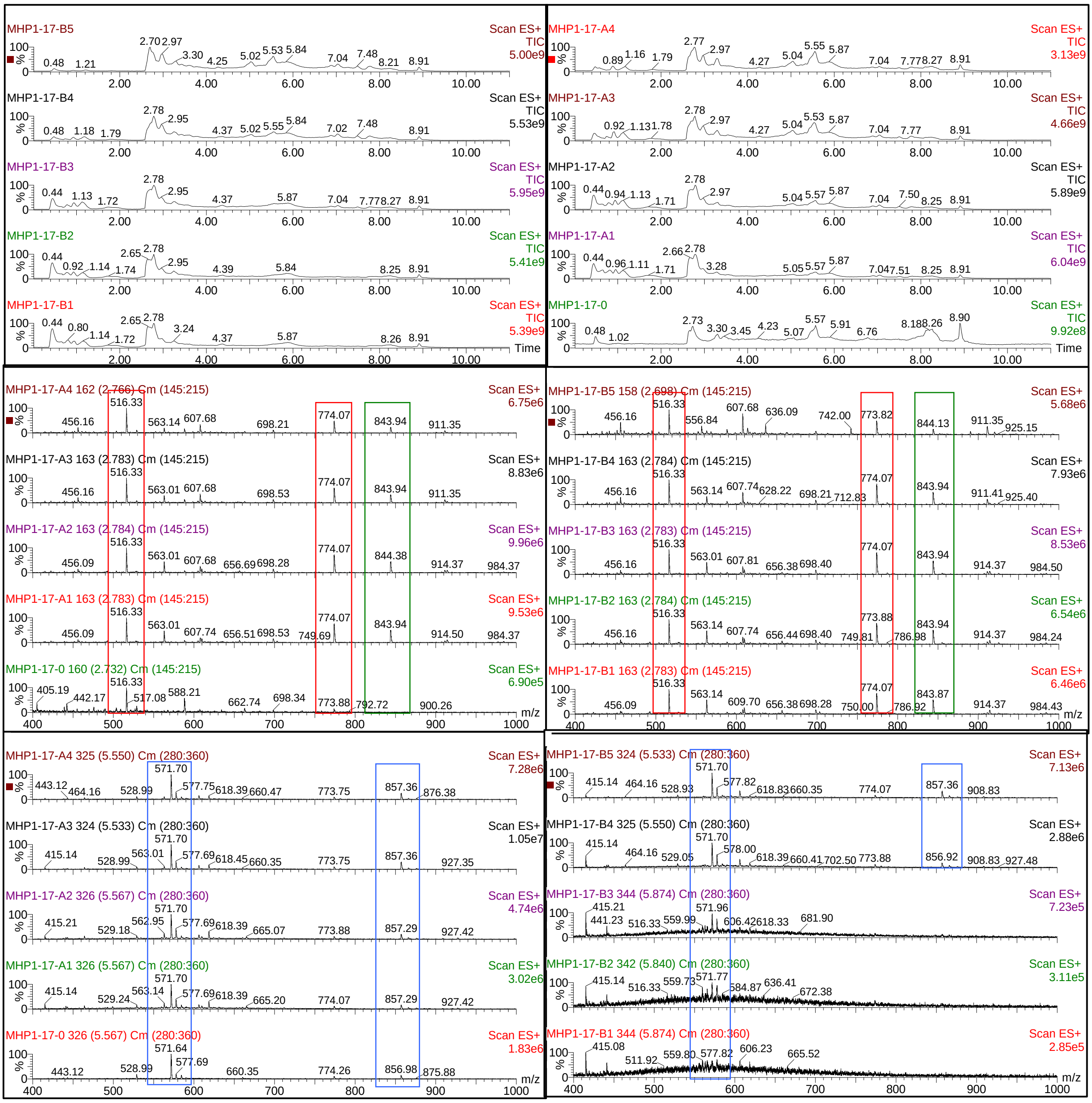

Figure S1. UPLC analysis of products obtained for Fur-GATCT-Gly-NH_2 PNA synthesis obtained under various conditions (A1–A4 and B1–B5 tests reported above) using *N*-(*N*-Boc-2-amminoethyl)maleimide as protecting group. Left column: tests A, reference; Right column: tests B. Top panels: UPLC-MS traces; middle panels: MS spectra of 2.40–3.30 minutes region; bottom panels: MS spectra of 5.00–6.00 minutes region. MW target (red boxes) = 1546.5; $[MH_2]^{2+}$ = 774.3; $[MH_3]^{3+}$ = 516.5; MW alkylated product = 1712,7 (blue boxes); $[MH_2]^{2+}$ = 857.4; $[MH_3]^{3+}$ = 571.9 MW cycloadduct = 1686,6 (green boxes); $[MH_2]^{2+}$ = 844.3 ; $[MH_3]^{3+}$ = 563.2.

Maleimide concentration and equivalents optimization

<u>General protocol</u>

1- Load a 200 μL Eppendorf with 0.2 μmol resin (approx. 1.86 mg);

2- Prepare desired solutions of N-(N-Boc-2-amminoethyl)maleimide, and transfer them to the Eppendorf tube;

3- Run each condition in triplicate;

4- Allow Diels-Alder reaction to proceed for 7.5 hours at 90°C

5- Transfer the resin beads in a 1 mL SPE tube equipped with PE frit and wash the resin with DMF/DCM;

6- Add 100 μL of a 10% m-cresol solution in TFA and allow to react for 1h 30 min;

7- Percolate the solution in a 2 mL Eppendorf tube, and wash the resin with 80 μL TFA;

8- Add 1.8 mL ethyl ether and allow to precipitate for 2h at −20 °C;

9- Recover the precipitate by centrifugation, and wash the pellet with ethyl ether;

10- Redissolve the dry pellet in 500 μL mQ, transfer in a 500 μL Eppendorf tube and heat for 5 hours at 90 °C;

11- Submit the samples to UPLC-MS analysis.

12- Data analysis

<u>Solution used</u>

Maleimide concentration	30 eq	20 eq	10 eq
0.5 M	\	Test B1	Test C1
0.25 M	Test A2	Test B2	Test C2
0.1 M	Test A3	Test B3	\

<u>Target identification</u>

PNA \ Charge	No charge	2+	3+	4+
Target	1546.5	774	516	387
Alkylated	1712.7	857	571	429
Cycloaddition by-prod	1686.6	844	563	422

<u>Data analysis</u>: For each single UPLC-MS trace three different XIC where obtained extracting the signal relative to the 2+ and 3+ m/z signals. Peaks were then integrated using MassLynx 4.0 algorithm. <u>Results</u>: 20 equivalent of maleimide at a concentration of 0.25 M gives the best results in term of by-product formation.

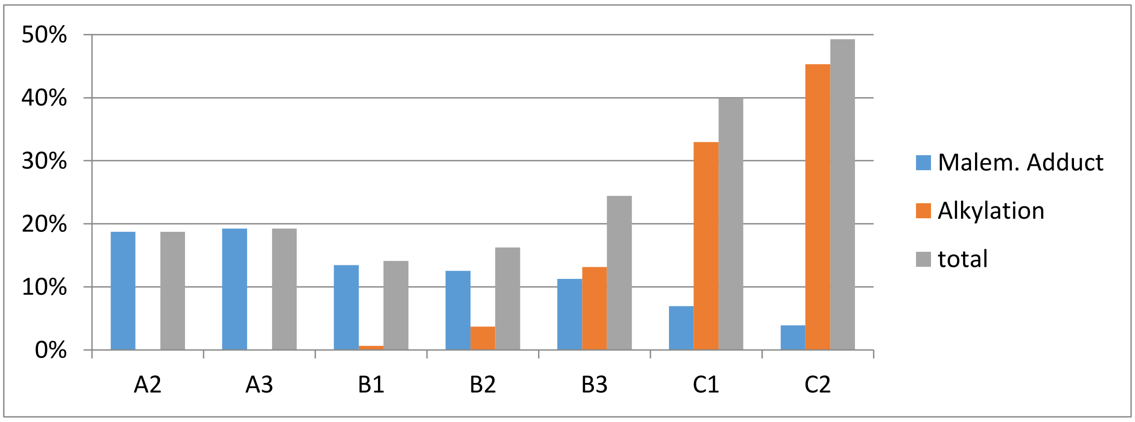

Figure S2. Relative by-product formation for Fur-GATCT-Gly-NH_2 PNA synthesis obtained under various conditions (A1–A4 and B1–B5 tests reported above) using *N*-(*N*-Boc-2-amminoethyl)maleimide as protecting group. Quantities are normalized to target product intensity in the UPLC-MS analysis (carried out as in figure S1). Data presented are an average of 3 different experiments.

Maleimide equivalents and reaction time optimization

General protocol

1- Load a 200 µL Eppendorf with 0.2 µmol resin (approx. 1.86 mg);
2- Prepare the desired 0.25 M solution of N-(N-Boc-2-amminoethyl)maleimide, and transfer the correct amount to the Eppendorf tube;
3- Run each condition in triplicate;
4- Allow Diels-Alder reaction to proceed for the specified time at 90°C
5- Transfer the resin beads in a 1 mL SPE tube equipped with PE frit and wash the resin with DMF/DCM;
6- Add 100 µL of a 10% m-cresol solution in TFA and allow to react for 1h30';
7- Percolate the solution in a 2 mL Eppendorf tube, and wash the resin with 80 µL TFA;
8- Add 1.8 mL ethyl ether and allow to precipitate for 2h at -20 °C;
9- Recover the precipitate by centrifugation, and wash the pellet with ethyl ether;
10- Redissolve the dry pellet in 500 µL mQ, transfer in a 500 µL Eppendorf tube and heat for 5 hours at 90 °C;
11- Submit the samples to UPLC-MS analysis.
12- Data analysis

Conditions used

Maleimide amount	15 h at 90°C	5 h at 90 °C
10 eq	Test A1	Test B1
20 eq	Test A2	Test B2
30 eq	Test A3	\

Target identification

PNA \ Charge	No charge	2+	3+	4+
Target	1546.5	774	516	387
Alkylated	1712.7	857	571	429
Cycloaddition by-prod	1686.6	844	563	422

Data analysis: For each single UPLC-MS trace three different XIC were obtained extracting the signal relative to the 2+ and 3+ m/z signals. Peaks were then integrated using MassLynx 4.0 algorithm.

Results: The use of 10 equivalents of maleimide at a concentration of 0.25 M gives the best results in terms of by-product formation, if the Diels-Alder reaction is conducted for a reduced time (5h).

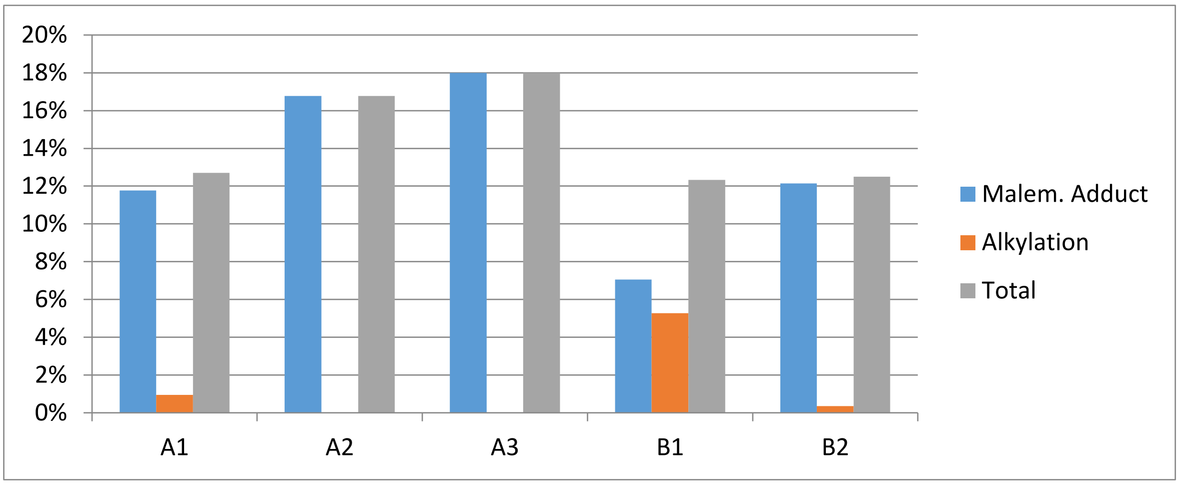

Figure S3. Relative by-product formation for Fur-GATCT-Gly-NH_2 PNA synthesis obtained under various conditions (A1–A4 and B1–B5 tests reported above) using *N*-(*N*-Boc-2-amminoethyl)maleimide as protecting group. Quantities are normalized to target product intensity in the UPLC-MS analysis (carried out as in figure S1). Data presented are an average of 3 different experiments.

Visualization of retro-DA reaction time course

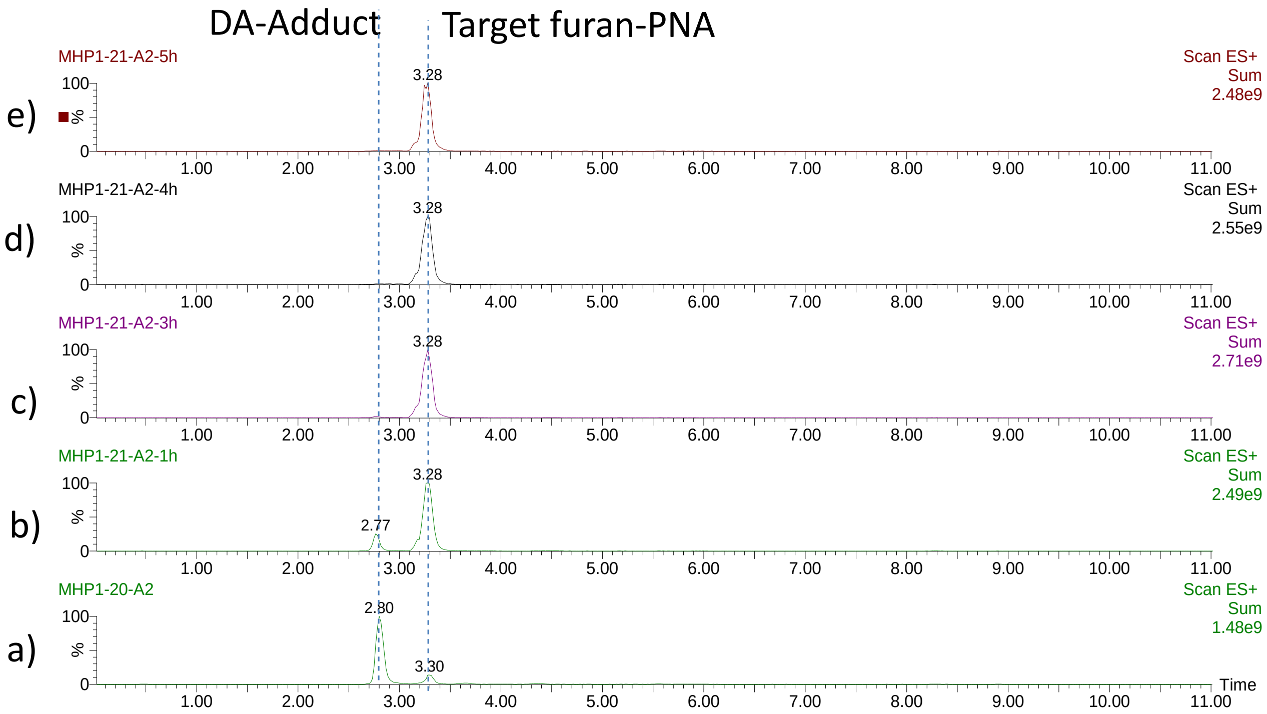

Figure S4. Time-course of the retro-DA reaction. UPLC-MS chromatograms (XIC with m/z selected for the Diels alder product (2.77 min) and for the Retro-Diels-Alder product (3.28 min) for reactions carried out for Fur-GATCT-Gly-NH_2 PNA synthesis obtained using 20 equivalents of N-(N-Boc-2-amminoethyl)maleimide in the protection step (7.5 h at 90°C) , and different times of the retro-DA reaction at 90°C after cleavage. a) before retro-DA; b-e: after 1, 3, 4, and 5 h respectively.

HPLC-DAD-HRMS chromatograms

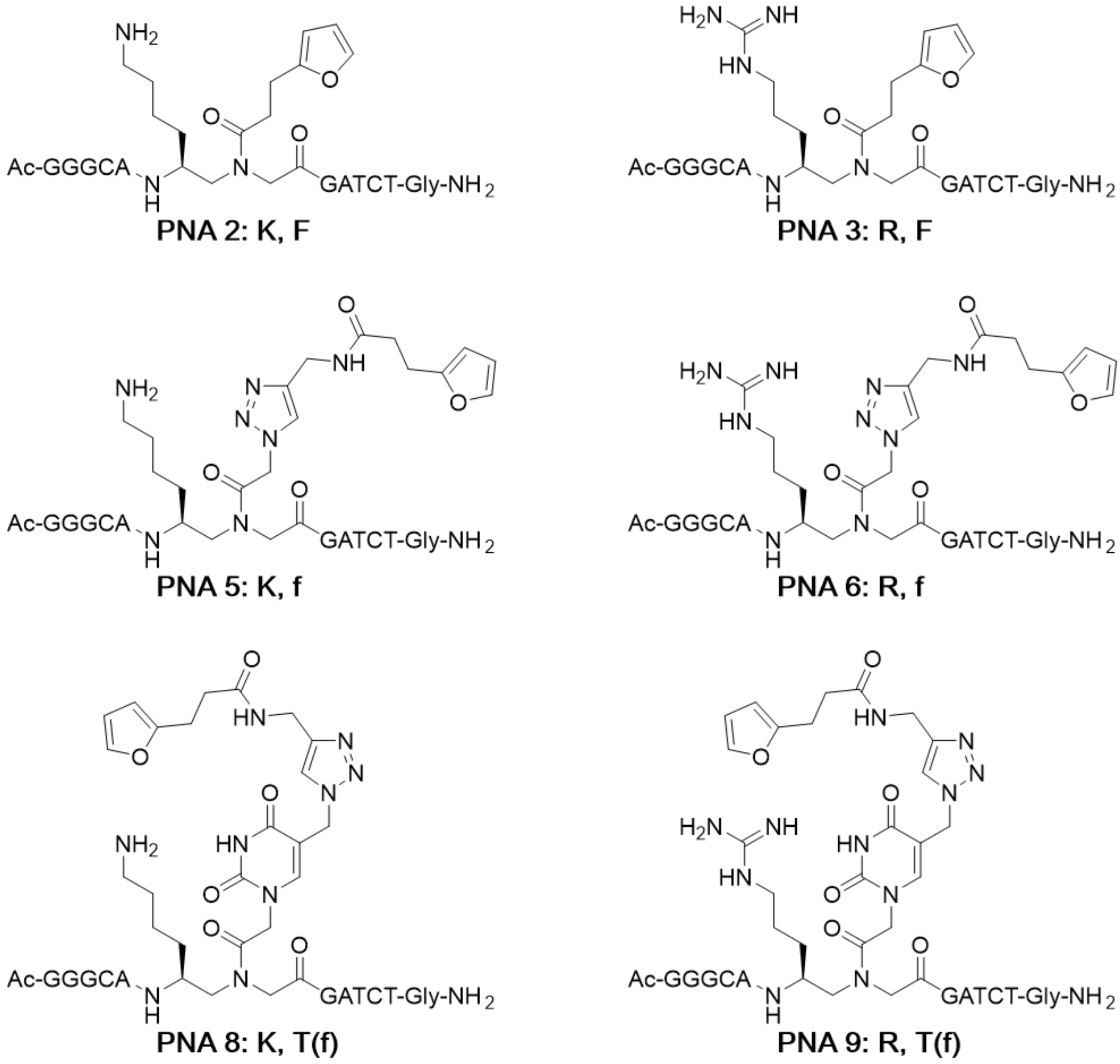

Figure S5. Overview of PNA sequences and furan-PNA monomers.

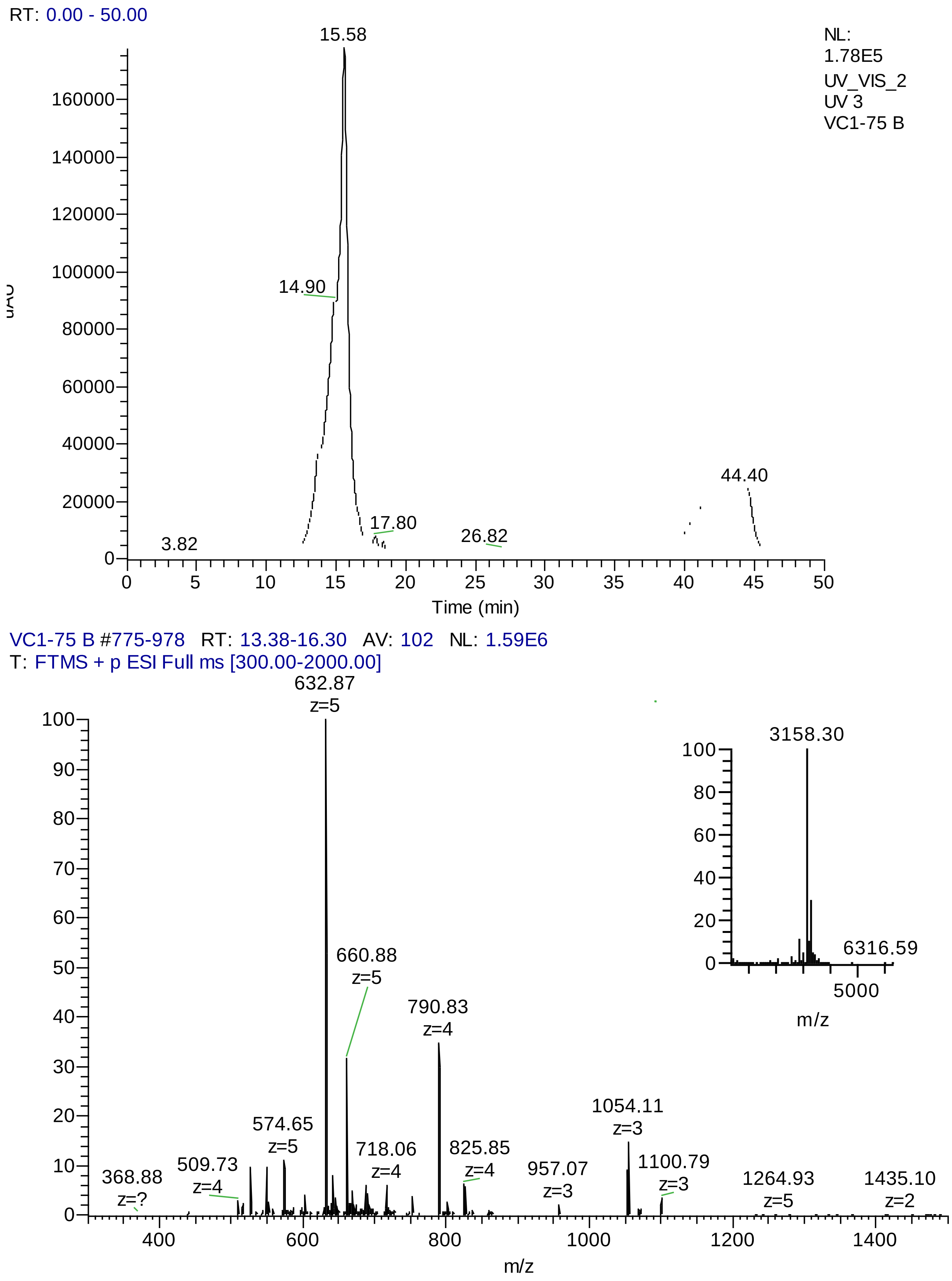

Figure S6. HPLC-DAD-HRMS chromatogram of purified **PNA 2**. HPLC-UV at 254 nm trace (top), MS spectrum of the corresponding peak at 15.58 min (center) and mathematical deconvolution of the multicharged signals (insert). (MW: 3158.2976; Molecular formula: $C_{132}H_{169}N_{69}O_{36}$).

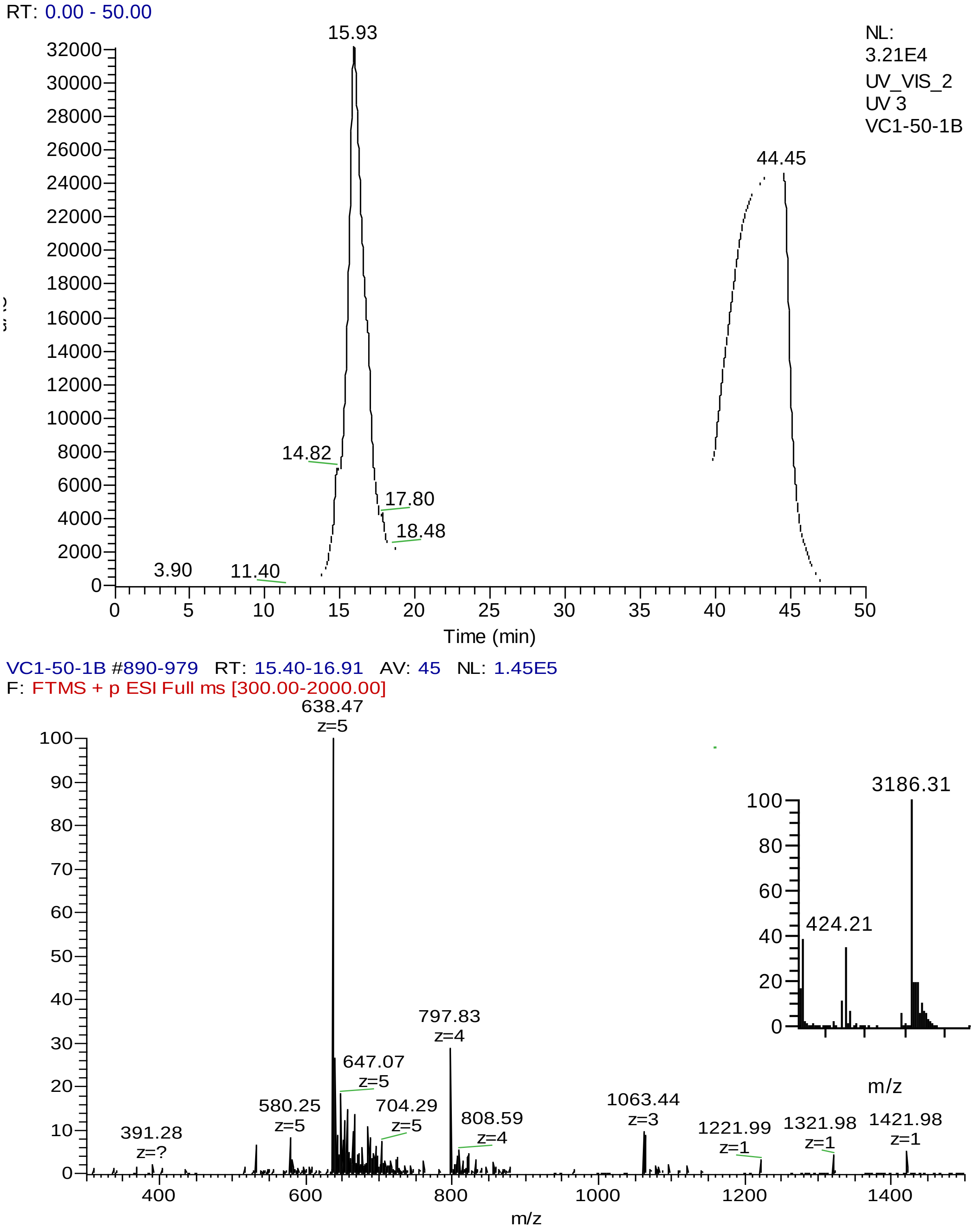

Figure S7. HPLC-DAD-HRMS chromatogram of purified **PNA 3**. HPLC-UV at 254 nm trace (top), MS spectrum of the corresponding peak at 15.93 min (center) and mathematical deconvolution of the multicharged signals (insert). (MW: 3186.3051; Molecular formula: $C_{132}H_{169}N_{71}O_{36}$).

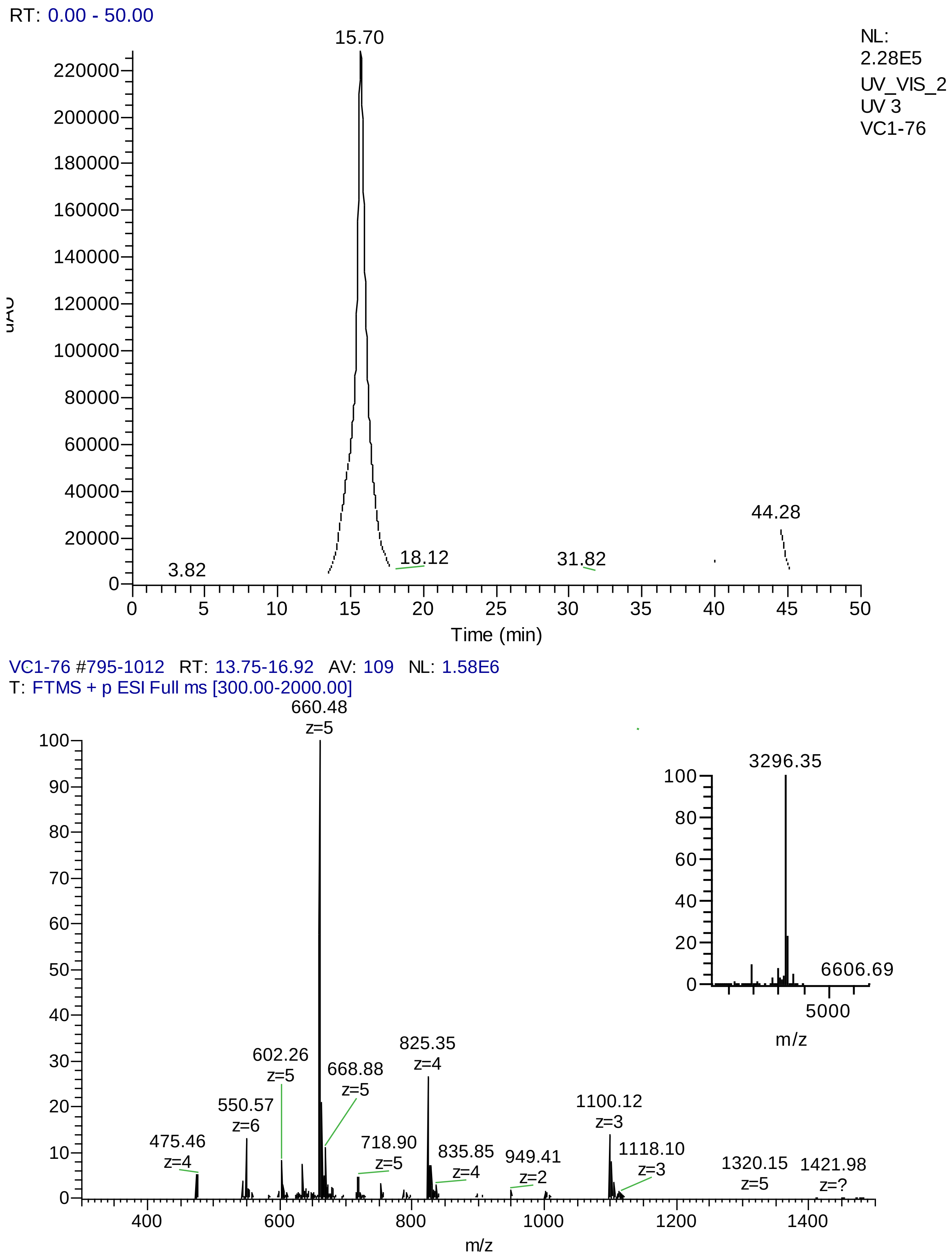

Figure S8. HPLC-DAD-HRMS chromatogram of purified **PNA 5**. HPLC-UV at 254 nm trace (top), MS spectrum of the corresponding peak at 15.70 min (center) and mathematical deconvolution of the multicharged signals (insert). (MW: 3296.3502; Molecular formula: $C_{127}H_{163}N_{65}O_{35}$).

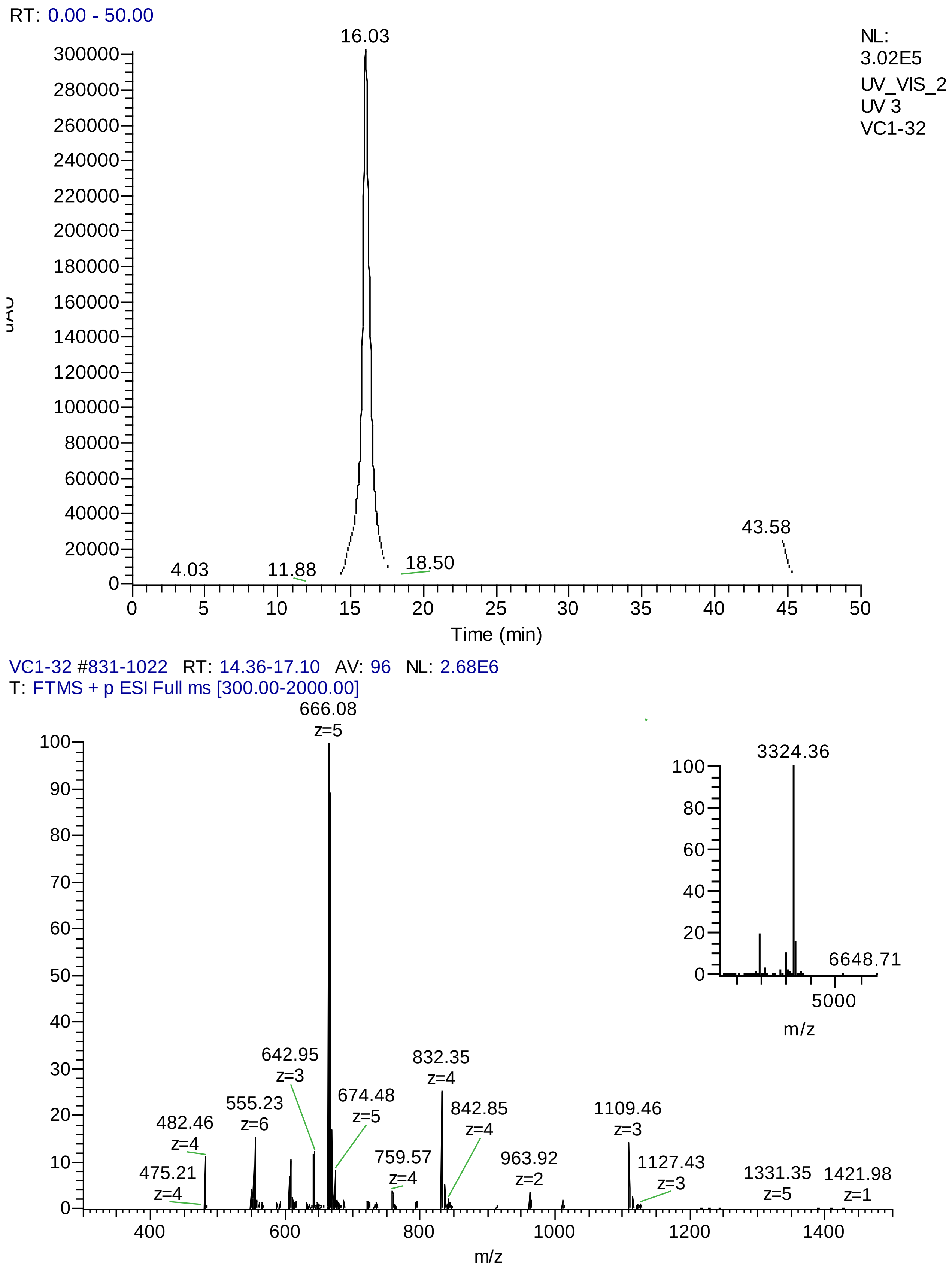

Figure S9. HPLC-DAD-HRMS chromatogram of purified **PNA 6**. HPLC-UV at 254 nm trace (top), MS spectrum of the corresponding peak at 16.03 min (center) and mathematical deconvolution of the multicharged signals (insert). (MW: 3324.3570; Molecular formula: $C_{127}H_{163}N_{67}O_{35}$).

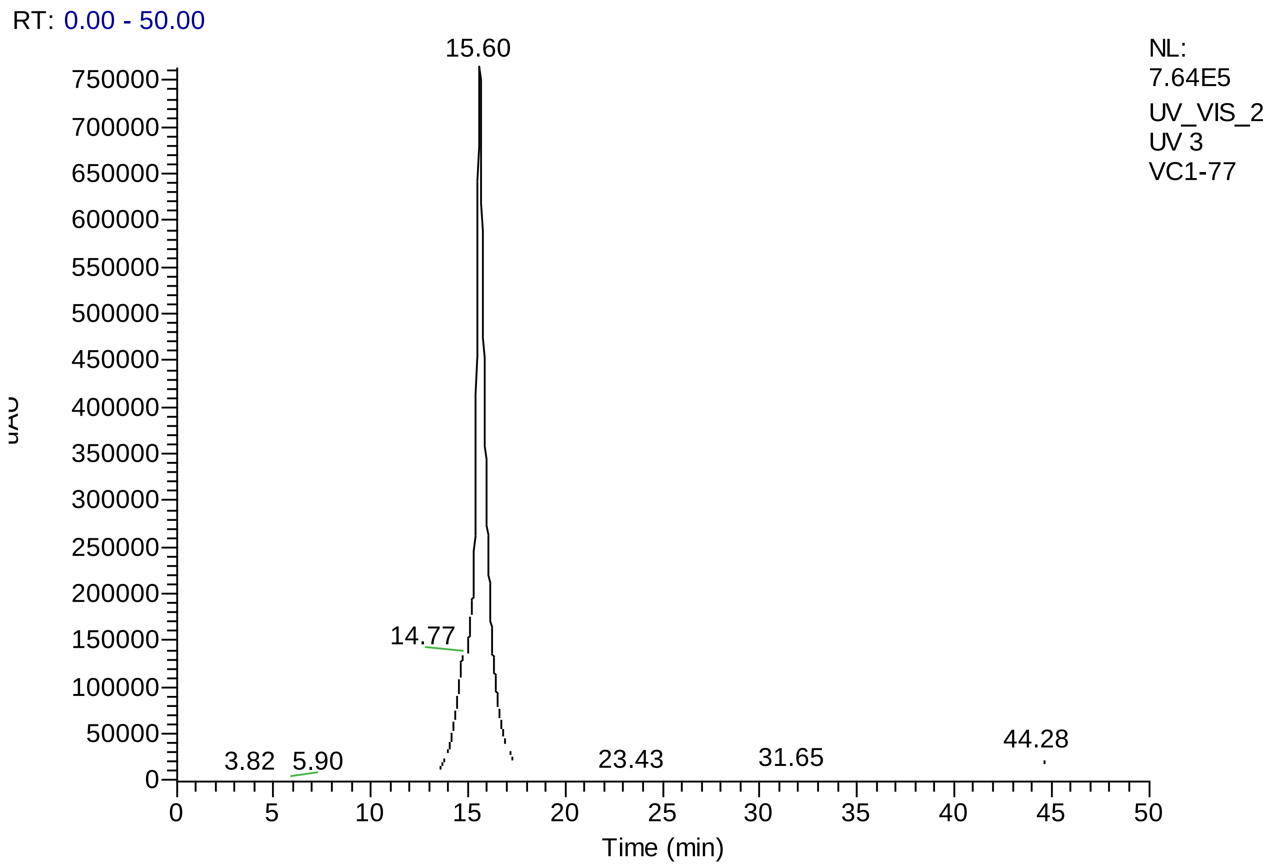

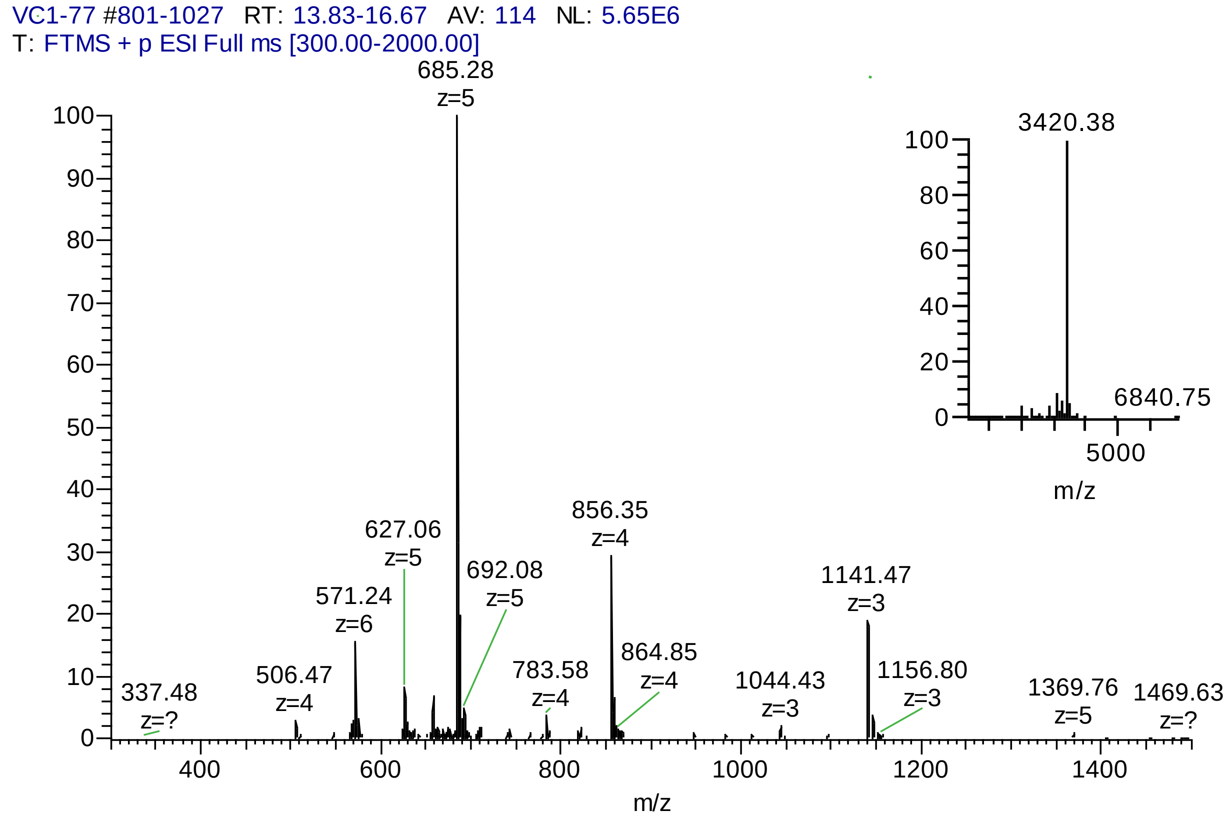

Figure S10. HPLC-DAD-HRMS chromatogram of purified **PNA 8**. HPLC-UV at 254 nm trace (top), MS spectrum of the corresponding peak at 15.60 min (center) and mathematical deconvolution of the multicharged signals (insert). (MW: 3420.3795; Molecular formula: $C_{137}H_{173}N_{71}O_{38}$).

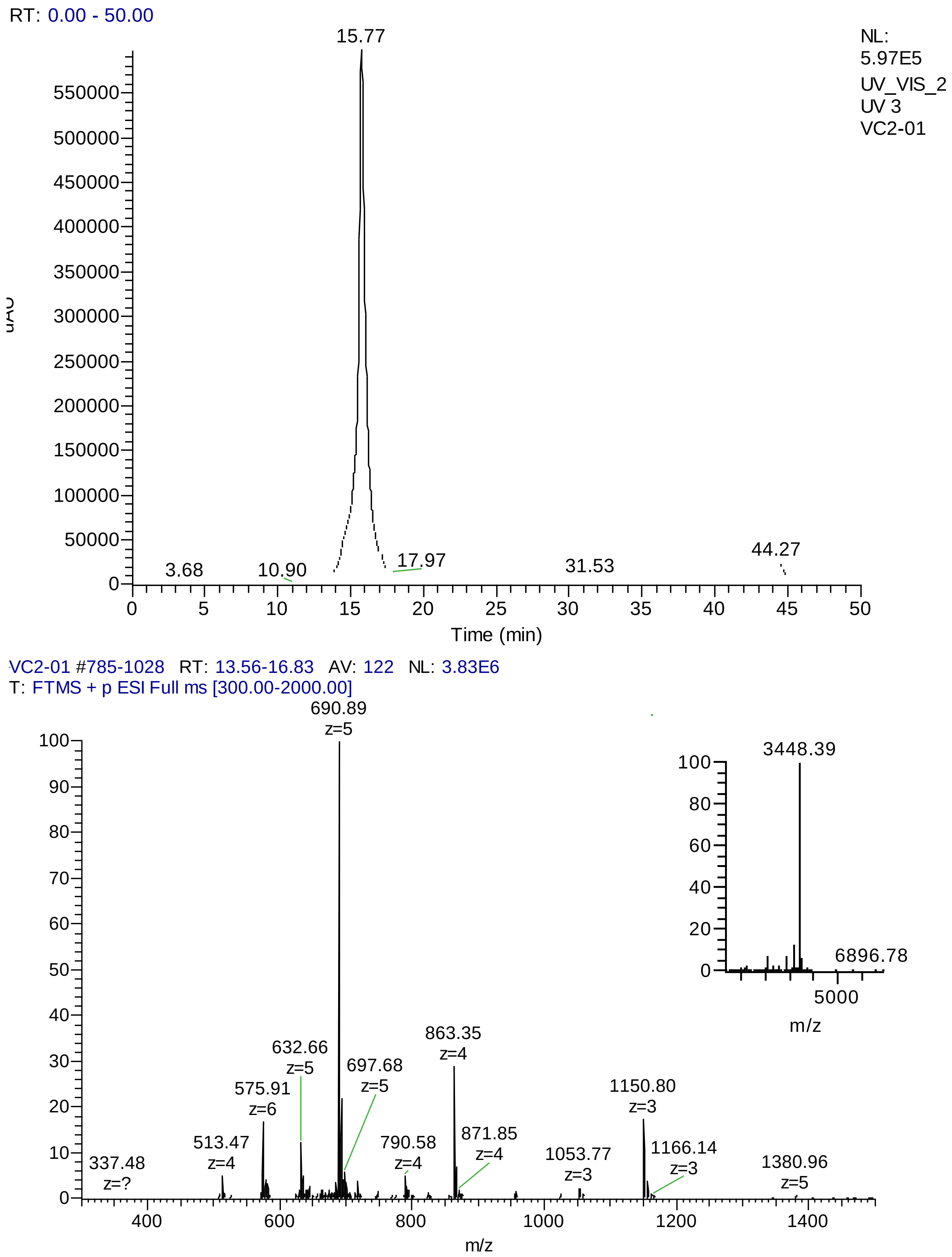

Figure S11. HPLC-DAD-HRMS chromatogram of purified **PNA 9**. HPLC-UV at 254 nm trace (top), MS spectrum of the corresponding peak at 15.77 min (center) and mathematical deconvolution of the multicharged signals (insert). (MW: 3448.3870; Molecular formula: $C_{137}H_{173}N_{73}O_{38}$).

PAGE analysis

Crosslink samples were analyzed on a 20% polyacrylamide gel (acrylamide:bisacrylamide 19:1) prepared in 1x Tris-Borat-EDTA (TBE) buffer containing 7 M urea. The temperature of the gel was stabilized with a Julabo F12 at 25°C. The power supply used for gel electrophoresis was a consort EV202 and a constant voltage of 230 V was used to run the gels. Gels were stained with SYBR gold (Thermo Fisher Scientific, Life Technologies) and pictures were taken with an Autochemi imaging system (UVP). 4 µL of the crosslink solution (10 µM) were mixed with 16 µL formamide and from this mixture 8 µL were loaded on the gel. It should be noted that staining of PNA is considerably more difficult and much less sensitive that DNA staining. As a result individual ssPNA strands cannot be visualized on PAGE and can thus not be seen in the figures below. Consequently also PNA-DNA crosslinked complexes (boxes in purple) are only visualized with low intensity.

A DNA impurity occurring in all samples appears in all gels as a slower running spot between the cross-linked complex and the ssDNA spots, due to the considerably higher sensitivity of DNA for SybrGold staining

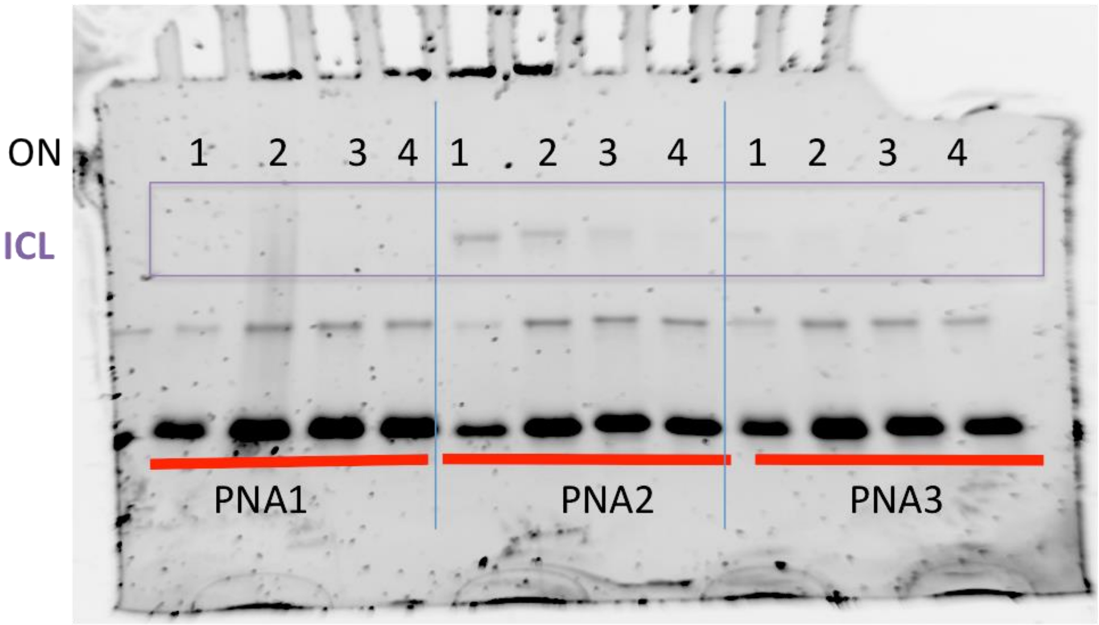

Figure S12. PAGE analysis: Cross-link experiment between **ON 1-4** and **PNA 1-3**. Analysis was done on a 20% polyacrylamide gel (acrylamide:bisacrylamide 19:1) prepared in a TBE buffer containing 7M urea. A constant voltage of 230V was applied at a temperature of 25 °C. Gels were stained with a SYBR gold solution and gels were analysed using an Autochemi Imaging system.

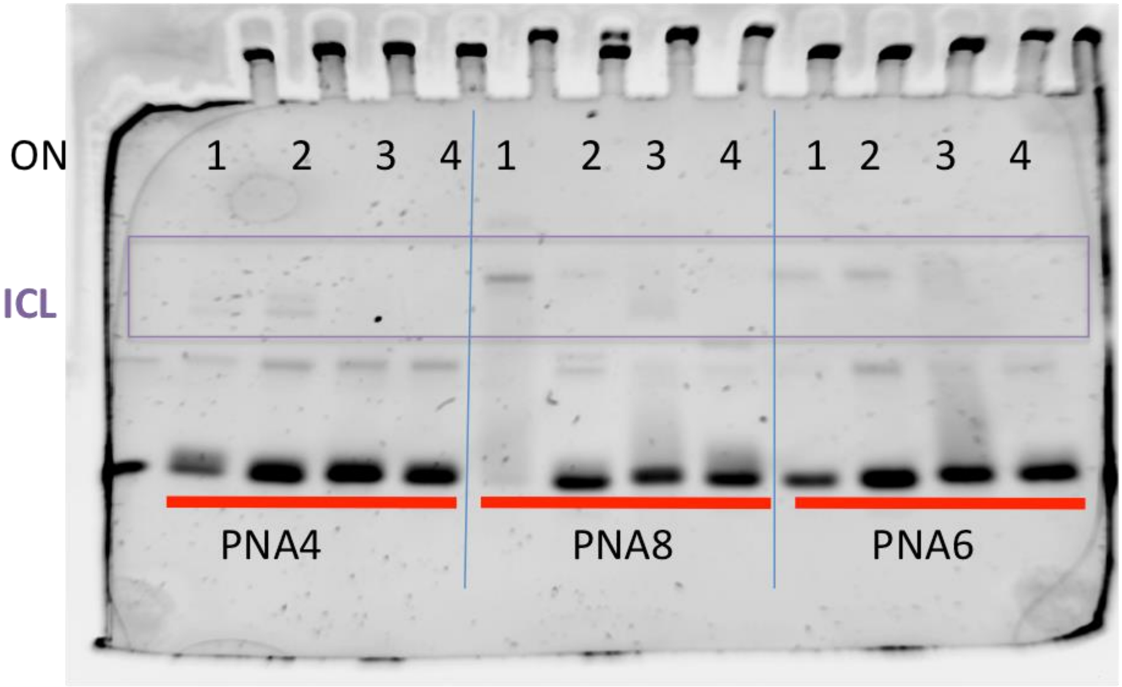

Figure S13. PAGE analysis: cross-link experiment between **ON 1**-**4** and **PNA 4**-**6**. Analysis was done on a 20% polyacrylamide gel (acrylamide:bisacrylamide 19:1) prepared in a TBE buffer containing 7M urea. A constant voltage of 230V was applied at a temperature of 25 °C. Gels were stained with a SYBR gold solution and gels were analysed using an Autochemi Imaging system.

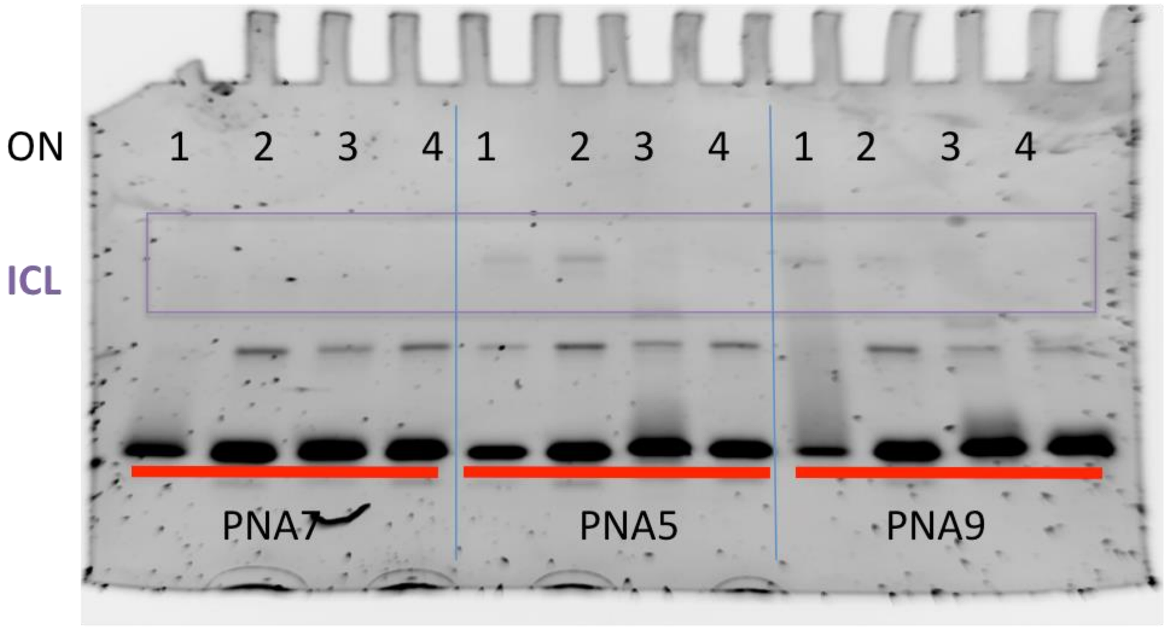

Figure S14. PAGE analysis: cross-link experiment between **ON 1**-**4** and **PNA 7**-**9**. Analysis was done on a 20% polyacrylamide gel (acrylamide:bisacrylamide 19:1) prepared in a TBE buffer containing 7M urea. A constant voltage of 230V was applied at a temperature of 25 °C. Gels were stained with a SYBR gold solution and gels were analysed using an Autochemi Imaging system.

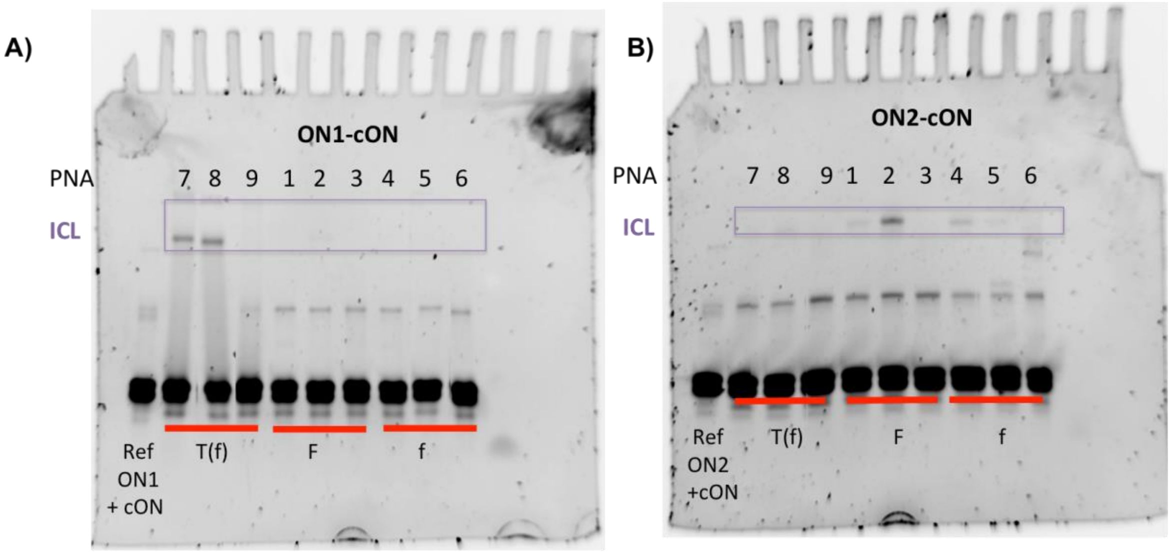

Figure S15. PAGE analysis. **A**) Strand displacement experiment of **ON1** and **PNA 1-9**. **B**) Strand displacement experiment between **ON 2** and **PNA 1-9**. Analysis was done on a 20% polyacrylamide gel (acrylamide:bisacrylamide 19:1) prepared in a TBE buffer containing 7M urea. A constant voltage of 230V was applied at a temperature of 25 °C. Gels were stained with a SYBR gold solution and gels were analysed using an Autochemi Imaging system.

MALDI analysis

Prior to MALDI analysis, the crosslinked product was purified using reverse phase HPLC (RP-HPLC). Purification was performed on an Agilent 1200b System equipped with a Waters X-bridge 130 Å Oligonucleotide C18 column (2.5 µM, 4.6 mm x 50 mm) at either a column temperature of 50 °C. Acetonitrile and 0.1 M TEAA buffer with 5% acetonitrile were used as mobile phase and were applied through a gradient of 0-18% MeCN in 20 minutes. The isolated crosslinked samples was dissolved in 5µL milliQ. Sample and matrix (2,5-dihydroxybenzoic acid; 15 mg in 100 µL milliQ and 50 µL MeCN with 1% TFA) were spotted in a 1:1 ratio onto the MALDI plate. The PNA-DNA crosslinked samples were analyzed by MALDI-TOF on an ABI Voyager DE-STR system equipped with a high performance nitrogen laser (337 nm). PNA-DNA crosslinked samples were analyzed in linear, positive mode.

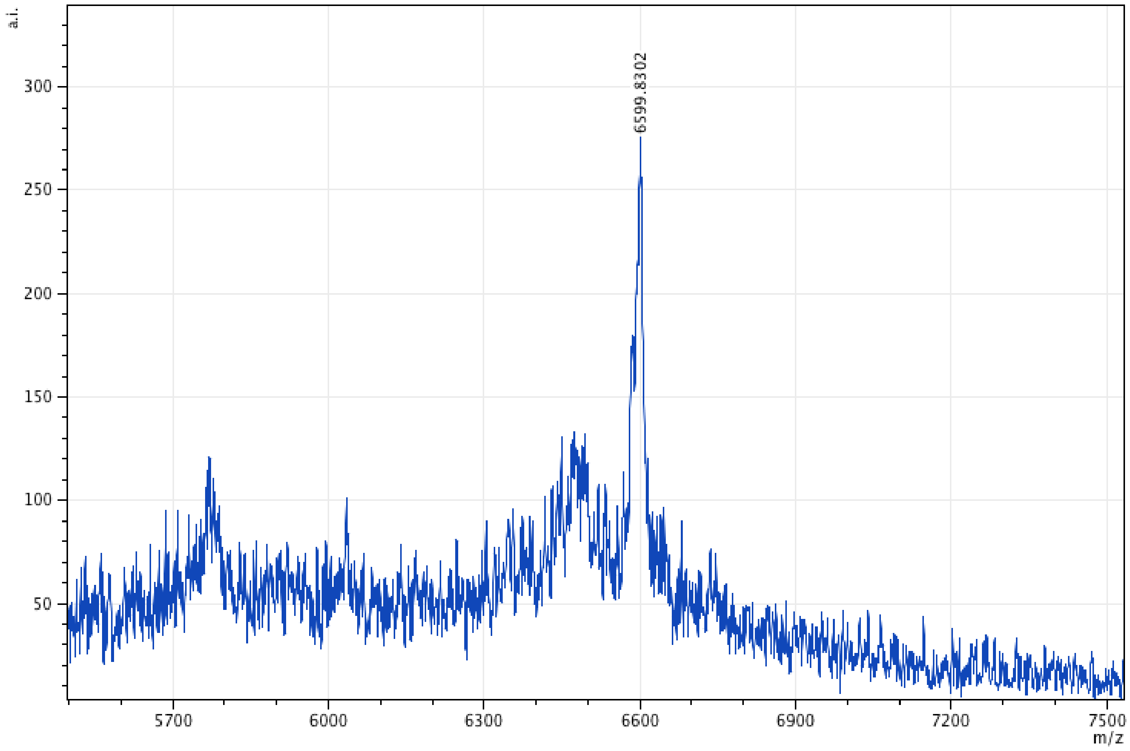

Figure S16. MALDI analysis of the cross-link product of **ON 2** and **PNA 4**. The labeled peak is attributed to the DNA-PNA cross-linked duplex (Expected mass: 6591 Da).

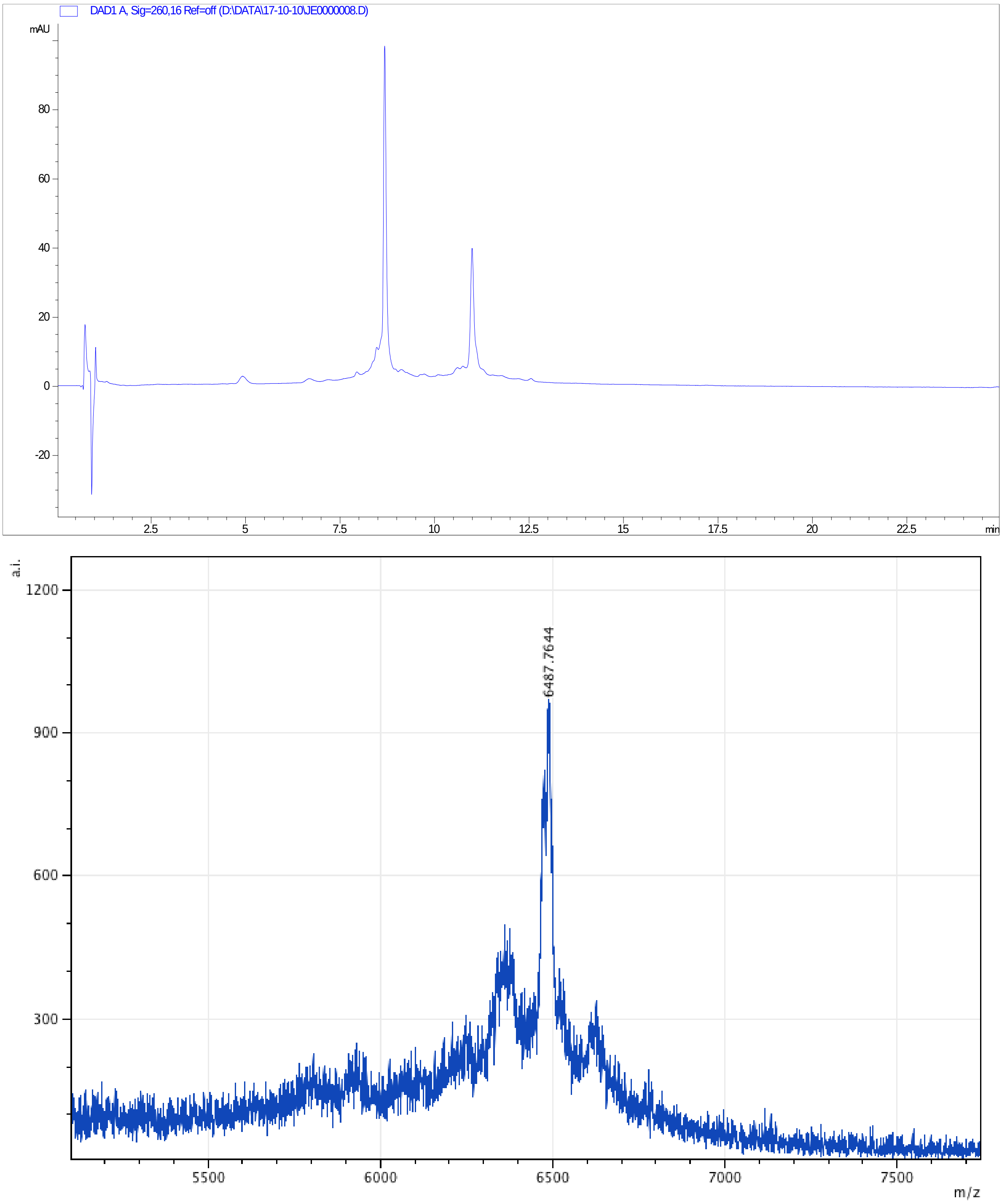

Figure S17. HPLC trace of the ICL experiment (top panel) and MALDI analysis (bottom panel) of the cross-link product of **ON 1** and **PNA 2** (peak at 11.00–11.50 min). The labeled peak is attributed to the DNA-PNA cross-linked duplex (Expected mass: 6477 Da). Peak at 8.5 min correspond to unreacted **ON 1**.

Mechanism of Interstrand Crosslink formation

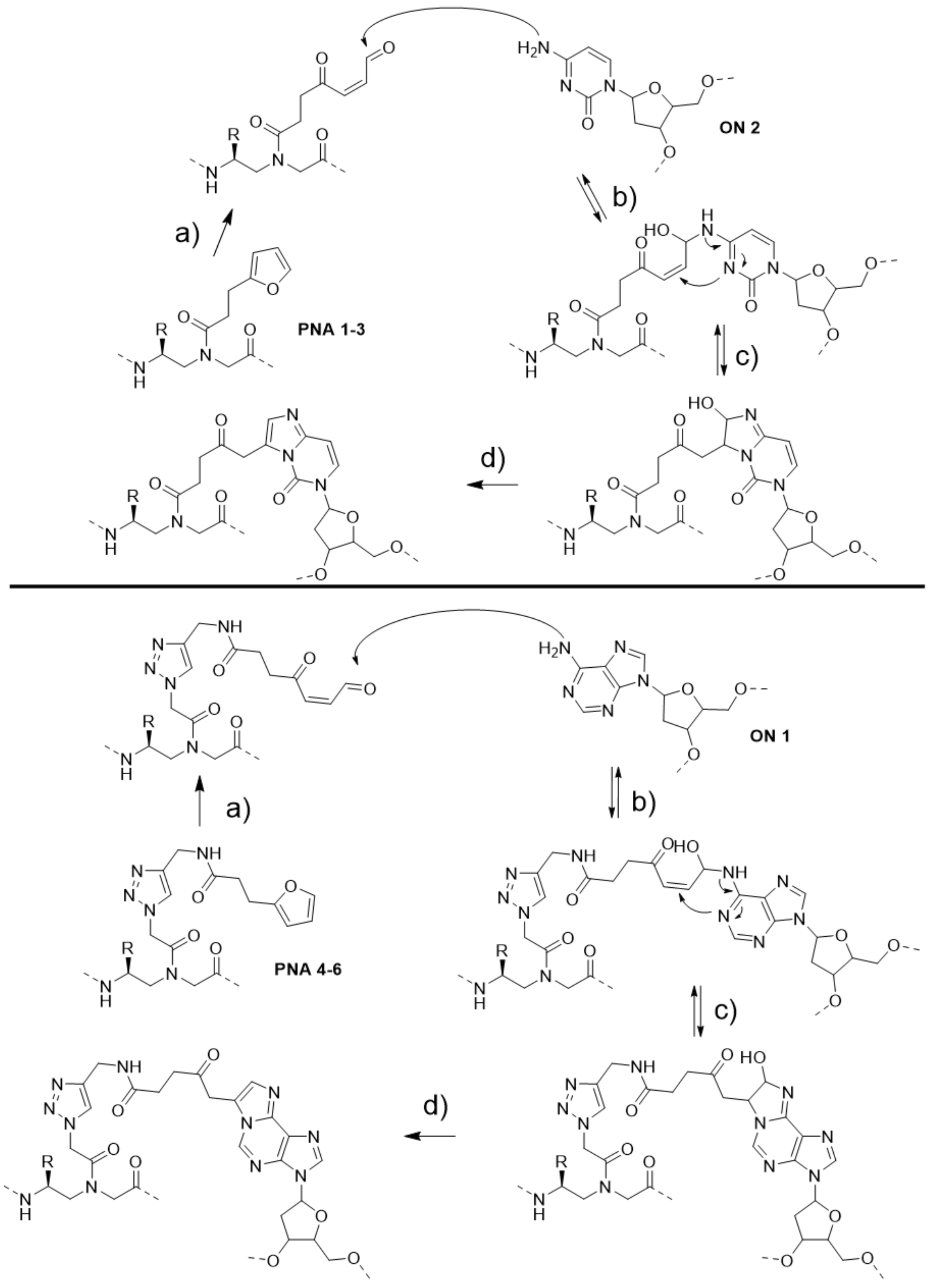

Figure S18. Schematic representation of interstrand crosslink formation between PNAs bearing **F** building block (**PNA 1**-**3**) and **ON 2** (upper panel) and PNAs bearing **f** building block (**PNA 4**-**6**) and **ON 1** (lower panel). a) Activation of the furan unit; b) reaction of the keto-enal with the exocyclic amine; c) ring closure reaction; d) re-aromatization reaction, through dehydration.
